# Intron retention in health and amyotrophic lateral sclerosis

**DOI:** 10.1093/brain/awag142

**Published:** 2026-08-03

**Authors:** Chloe Y Wang, Stephanie Taylor, Virenkumar A Pandya, Benjamin E Clarke, Koustav Pal, Tatyana A Shelkovnikova, Yiran Wang, Raphaëlle Luisier, Rickie Patani

**Affiliations:** The University of California, Los Angeles, CA 90095, USA; Department of Neuromuscular Diseases, Queen Square Institute of Neurology, University College London, London WC1N 3BG, UK; Laboratory for Human Stem Cells and Neurodegeneration, The Francis Crick Institute, London NW1 1AT, UK; Department of Neurodegenerative Disease, Queen Square Institute of Neurology, UCL, London WC1N 3BG, UK; Royal Free London NHS Foundation Trust, London NW3 2QG, UK; Department of Neuromuscular Diseases, Queen Square Institute of Neurology, University College London, London WC1N 3BG, UK; Laboratory for Human Stem Cells and Neurodegeneration, The Francis Crick Institute, London NW1 1AT, UK; Neurobiology Programme, Life Sciences Institute, Centre for Life Sciences, National University of Singapore, 117456 Singapore, Singapore; Department of Medicine, Yong Loo Lin School of Medicine, National University of Singapore, 119228 Singapore, Singapore; Department of Anatomy, Yong Loo Lin School of Medicine, National University of Singapore, 117594 Singapore, Singapore; Department of Neuromuscular Diseases, Queen Square Institute of Neurology, University College London, London WC1N 3BG, UK; Laboratory for Human Stem Cells and Neurodegeneration, The Francis Crick Institute, London NW1 1AT, UK; Neurobiology Programme, Life Sciences Institute, Centre for Life Sciences, National University of Singapore, 117456 Singapore, Singapore; Department of Medicine, Yong Loo Lin School of Medicine, National University of Singapore, 119228 Singapore, Singapore; Department of Anatomy, Yong Loo Lin School of Medicine, National University of Singapore, 117594 Singapore, Singapore; Sheffield Institute for Translational Neuroscience (SITraN) and Neuroscience Institute, University of Sheffield, Sheffield S10 2HQ, UK; Department of Neuromuscular Diseases, Queen Square Institute of Neurology, University College London, London WC1N 3BG, UK; Laboratory for Human Stem Cells and Neurodegeneration, The Francis Crick Institute, London NW1 1AT, UK; Oxford Motor Neuron Disease Centre, Nuffield Department of Clinical Neurosciences, University of Oxford, John Radcliffe Hospital, Oxford OX3 9DU, UK; Kavli Institute for Nanoscience Discovery, University of Oxford, Oxford OX1 3QU, UK; Swiss Institute of Bioinformatics, Lausanne 1015, Switzerland; Department for BioMedical Research, University of Bern, Bern 3008, Switzerland; Department of Neuromuscular Diseases, Queen Square Institute of Neurology, University College London, London WC1N 3BG, UK; Laboratory for Human Stem Cells and Neurodegeneration, The Francis Crick Institute, London NW1 1AT, UK; Department of Neurodegenerative Disease, Queen Square Institute of Neurology, UCL, London WC1N 3BG, UK; Neurobiology Programme, Life Sciences Institute, Centre for Life Sciences, National University of Singapore, 117456 Singapore, Singapore; Department of Medicine, Yong Loo Lin School of Medicine, National University of Singapore, 119228 Singapore, Singapore; Department of Anatomy, Yong Loo Lin School of Medicine, National University of Singapore, 117594 Singapore, Singapore; Neuroscience Translational Research Programme, Yong Loo Lin School of Medicine, National University of Singapore, 119228 Singapore, Singapore

**Keywords:** intron retention, amyotrophic lateral sclerosis, gene expression regulation, RNA binding protein, splicing, phase separation

## Abstract

Intron retention (IR) is the molecular phenomenon by which introns, historically thought to represent non-coding ‘junk’, remain unspliced within pre-mRNA transcripts, resulting in their incorporation into the mature mRNA molecule. While the role of IR is well established in species of plant, fungi, insects and viruses, it remains relatively understudied in mammalian biology. It was previously assumed that IR only played a limited role in downregulating a transcript’s translation potential through downstream initiation of nuclear detention or nonsense mediated decay (NMD). However, recent studies highlight IR’s significantly more complex and dynamic contribution to cellular physiology and disease. In particular, a role for IR is emerging in both health and neurodegenerative diseases, including amyotrophic lateral sclerosis (ALS), a rapidly progressive and invariably fatal disease that renders patients paralysed and unable to eat, speak or breathe. Significant technological advances now permit a comprehensive interrogation of previously unrecognized aspects of RNA metabolism in clinically relevant human cell types.

In this review, we focus on the differential role(s) of nuclear and cytoplasmic intron retaining transcripts (nIRTs and cIRTs, respectively), as well as how IRTs may influence subcellular localization of ribonucleoprotein (RNP) complexes, loss of function of bound RNA binding proteins (RBPs) and liquid-liquid phase separation (LLPS) in physiology and disease. Additionally, we discuss the potential of IRTs as independent regulatory elements beyond their protein-coding functions and highlight how artificial intelligence is poised to accelerate discoveries in this area. In the context of IR’s increasing appreciation, we also highlight its potential as a therapeutic target and explore current and future challenges in this burgeoning field.

## Teaser

During gene expression, RNA transcripts are typically spliced to remove introns before nuclear export and protein synthesis. Wang *et al.* examine intron retention—an alternative splicing event increasingly implicated in ALS—and discuss how retained introns influence RNA metabolism, protein regulation, and neurodegeneration.

## Introduction

Neurodegenerative disorders are often taxonomized as being predominantly protein aggregation disorders (e.g. Alzheimer’s disease or AD) or those related to defects in RNA metabolism (e.g. amyotrophic lateral sclerosis or ALS). However, this is potentially a facile dichotomy, given that both processes are fundamentally implicated in different forms of neurodegeneration and this is certainly the case in ALS, a rapidly debilitating and universally fatal neurodegenerative disorder primarily involving motor neurons (reviewed elsewhere).^1^ The common molecular denominator of these implicated pathogenic processes is the ribonucleoprotein (RNP) complex, composed of RNA and RNA binding proteins (RBPs), which plays a multilayered role in the regulation of gene expression.^2^

Indeed, it has been two decades since TAR DNA binding protein 43 (TDP-43) protein was discovered as the major constituent of ubiquitinated cytoplasmic inclusions in the vast majority of ALS and about 50% of frontotemporal dementia (FTD) cases.^3^ This finding galvanized significant research focus towards TDP-43 and its interactome for both mechanistic discovery and therapeutic target development. TDP-43 is an RBP and a DNA binding protein. It predominantly resides in the nucleus and plays key roles in pre-RNA splicing, mRNA stabilization, RNA transport, DNA repair mechanisms and biocondensate formation.^4^ In physiology, TDP-43 shuttles to the cytoplasm and contributes to the fate of processed RNA targets. TDP-43’s mislocalization, phosphorylation, cleavage and aggregation in the cytoplasm with nuclear depletion is a hallmark feature of ALS motor neurons, and has been recently reviewed comprehensively in this context.^5^ Given the dearth of life-extending options for ALS patients, a deeper mechanistic understanding of how deregulated RNA metabolism precisely contributes to neurodegeneration remains a key priority, with the potential to yield novel therapeutic opportunity and clinical impact.

**Figure 1:**
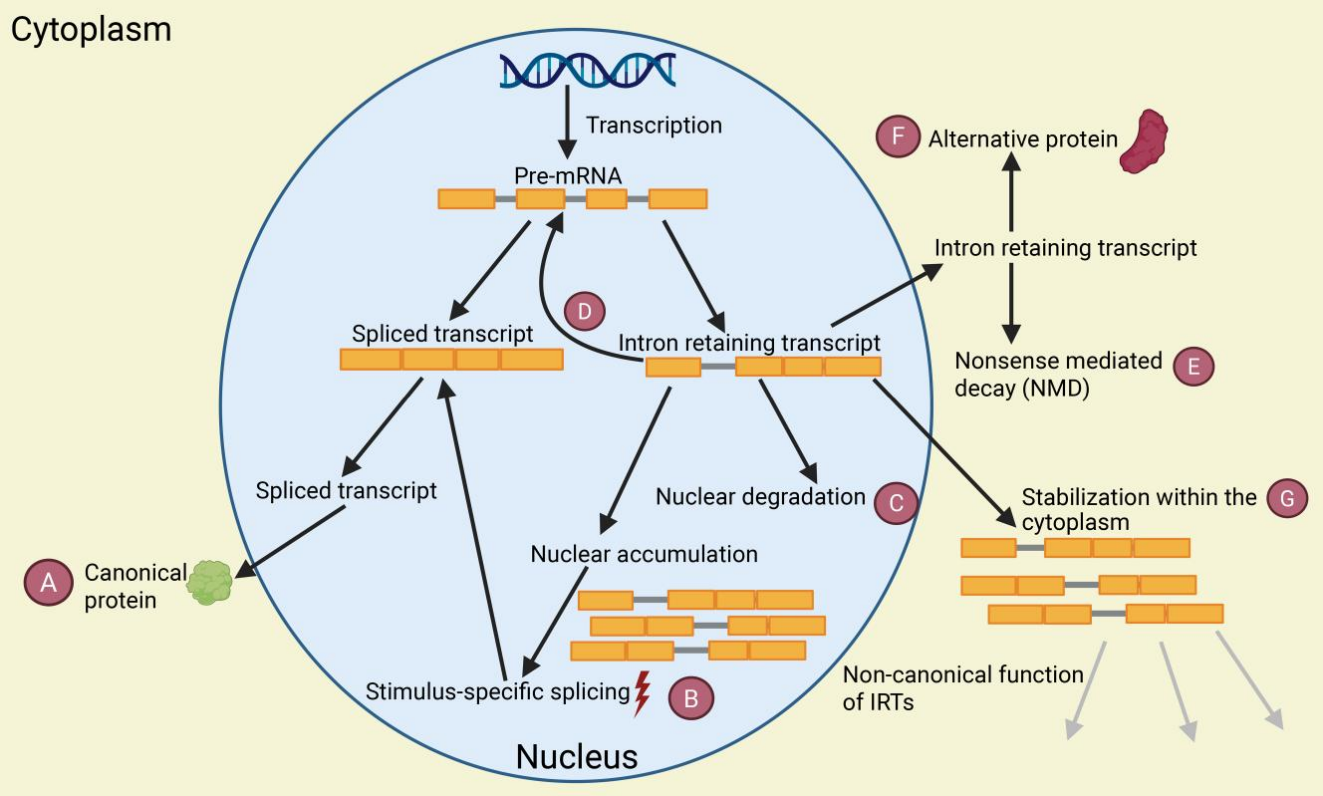
**The retention of an intronic sequence with mRNA can lead to multiple fates.** Canonically spliced mRNA is typically exported to the cytoplasm for translation (**A**). However, intron retaining transcripts (IRTs) may be stored or ‘poised’ in the nucleus and only spliced following a specific stimulus (**B**), after which they feed into the canonical pathway (**A**) for translation. IRTs may also be degraded in the nucleus by the exosome (**C**) or play a role in cotranscriptional splicing regulation (**D**). If the IRT is exported to the cytoplasm, it may be degraded by nonsense-mediated mRNA decay (NMD; **E**), non-canonically translated into a (likely truncated) protein (**F**) or stabilized within the cytoplasm (**G**). Created in BioRender. Wang, C. (2026) https://BioRender.com/oxkr523.

The canonical information ‘flow’ from DNA through messenger RNA (mRNA) to protein (Fig. 1A) necessitates a series of increasingly understood, albeit complex, events. In general, following transcription, pre-mRNA undergoes 5′-end capping, splicing, 3′-end cleavage and polyadenylation, following which mature mRNA is exported to the cytoplasm, localized, translated, stabilized or degraded. These processes are regulated by RBPs and non-coding RNAs such as microRNAs (miRNAs).

RBPs are one of the largest groups of proteins: exact estimates of the number of RBPs depends on methodology used, but can confidently be estimated at over 3400, and may be over 4200 when integrating high-throughput RBP detection studies in different human cell types.^6^ It is likely that further proteins will be identified as RNA binding and regulating, with Cys2-His2 zinc-finger (C2H2-ZNF) transcription factors as recent examples.^7^ RBPs exhibit high translational efficiency, protein stability and abundance compared with other protein classes.^8^ In addition, approximately 2000 genes encode miRNAs, with many of these expressed from the non-protein-coding genome.^8-10^ These studies cumulatively highlight the enormous regulatory complexity of RNAs.

As part of the canonical splicing process, approximately 75% of introns are removed cotranscriptionally, i.e. during transcription;^11,12^ the remainder of splicing occurs post-transcriptionally. In particular, introns that flank alternative exons are sometimes excised post-transcriptionally.^13,14^ The human genome has the capacity to diversify the output of transcription through a process known as alternative splicing (AS). Intriguingly, transcripts from >90% of human genes undergo AS, where coding and non-coding fragments are alternatively skipped and joined.^15,16^ In most cases, this splicing out of introns ensures the production of appropriate protein isoforms in the correct cellular environments. In a form of AS however, namely intron retention (IR), the mature, polyadenylated mRNA transcript retains one or more introns. This process has recently gained much attention in mammalian biology. In the context of disease, it is conceivable that changes in the expression of subsets of increased intron-retaining transcripts (IRTs) observed in neurons and other CNS cell types represent either pathogenic or compensatory mechanisms, or have biomarker value, which further reinforces the need to investigate this relatively neglected form of AS to a greater degree.

## The established roles of intron retention: regulation of gene expression and beyond

Historically, IR has been studied extensively in viruses, fungi and plants but its prevalence in mammalian physiology has only been appreciated over the last two decades. Indeed, one study found that transcripts from approximately three-quarters of human genes contain at least one retained intron, with IR occurring in a cell type-dependent manner.^17^ It was found that IR was broadly functioning to reduce the level of transcripts where and when they are not required.^17^ Given the sheer number of IRTs, these data have challenged the long-held view that IR merely represents ‘transcriptional noise’. IR is now increasingly recognized to be a regulated process (elaborated in Box 1), influencing gene expression in a variety of ways and this has been reviewed comprehensively elsewhere.^18-20^ Intronic sequences, like other non-coding regions of mRNA such as the 3′ untranslated region (UTR), have traditionally been studied for their regulatory roles in association with protein-coding transcripts. However, as early as 1994, it was proposed that RNAs derived from introns could have evolved functional roles in the cell.^21^ Since then, growing evidence suggests that introns and other non-coding sequences, which make up over 90% of pre-mRNA, also possess independent regulatory and catalytic functions. Intronic sequences, for example, can generate stable RNA fragments that persist in nuclear and cytoplasmic compartments. The parallels that exist between intron retention and 3′UTRs are summarized in Box 2.

Box 1 Regulation of intron-retaining transcripts

Differential GC content between introns and exons has been posited to be a salient factor in spliceosomal recognition.^22^ In this model, introns with relatively high GC content use a mode of splicing that involves intron definition rather than the canonical exon definition. Higher GC content may lead to an increase in the complexity of secondary structures, which in turn can impact splice site accessibility. Intron definition refers to the recognition of splice signals within introns. In the context of an ‘intron definition’ mode of splicing, impaired splicing efficiency is more likely to result in an intron-retaining transcript (IRT) rather than skipped exons. Retained introns are generally shorter, flanked by weaker 5′ and/or 3′ splice sites and more evolutionarily conserved than constitutively spliced introns.^17,23^ It is noteworthy that this ‘IR code’ of cis-features has largely only been considered in the context of whole cell studies rather than compartment-specific interrogation, leaving the issue of whether nuclear versus cytoplasmic IRTs might have divergent IR codes unresolved.

Box 2 Noteworthy parallels between intron retention and 3′-untranslated regions

Non-canonical roles for RNAs are precedented. Prior research has, for example, revealed an unexpected function of the non-coding portion of an mRNA transcript independent of its translation potential, hinting that understanding the full range of regulatory functions of mature polyadenylated transcripts, of which intron retaining transcripts (IRTs) are one subset, remains an active area of discovery. One example is Tp53inp2 transcripts, which were shown to regulate axon growth independently of translation in sympathetic neurons, owing to Tp53inp2’s unusually long 3-untranslated region (UTR). In non-neuronal cells however, the transcript is canonically translated, demonstrating cell type-specificity in this role.^24^ Like intronic sequences, 3′ UTRs can also generate stable RNA fragments that persist in nuclear and cytoplasmic compartments. Stable 3′ UTR cleavage fragments have been detected in neurons,^25,26^ though their precise functions remain largely unknown.^26,27^ Beyond gene expression regulation, 3′ UTRs can also contribute to subcellular compartmentalization. 3′ UTRs can promote the formation of cytoplasmic, membraneless organelles,^28^ creating specialized environments for protein translation with direct functional consequences.^29,30^ Moreover, the striking similarities among intronic sequences, 5′ UTRs and 3′ UTRs, beyond shared sequence features like enrichment for RNA binding proteins and micro RNA binding sites, highlight the need to consider these regions as interconnected rather than distinct entities. Recognizing their shared properties should inspire broader investigations, where discoveries in one region can guide and stimulate similar explorations in the others, ultimately uncovering new regulatory and functional roles.

A little over a decade ago ‘stable intronic sequence RNAs’ or sisRNAs were identified in the *Xenopus* oocyte and described as being derived from excised intronic sequences.^31-35^ These were found to be surprisingly stable (up to 48 h), and abundant in the nucleus. More recently, there has been some convergence in nomenclature and the term ‘sisRNAs’ has been revised to encompass both some stable IRTs as well as excised introns, belonging to a broader class of long non-coding RNAs (lncRNAs). SisRNAs contribute to various cellular processes, including host gene regulation, acting as molecular sinks or sponges, and influencing protein translation.^32,35,36^ Notably however, while stable intron lariats are exported from the nucleus to the cytoplasm in a regulated manner,^35^ their excessive accumulation is detrimental to the cell.^37,38^ It is intriguing to contemplate whether these intronic fragments also possess regulatory or catalytic functions in essential biological processes, akin to other non-coding RNAs whose functional repertoires continue to evolve.^39,40^ Indeed, many lncRNAs originate from intronic regions, and some retain sequence features reminiscent of spliced-out introns.^41,42^

Thus, the idea that non-coding portions of mRNA such as introns have only limited regulatory roles which relate to their associated proteins is increasingly being challenged, highlighting the need to explore their full potential, paralleling the expanding roles of non-coding RNAs more generally. Many fundamental processes including cell cycle progression,^43^ cellular differentiation,^44^ activation of specific cellular substates,^45^ apoptosis^46^ and stress responses^47^ are now known to relate to changes in IR of particular subsets of functionally related transcripts. The repertoire of IR is cell type- and state-specific, and most frequently observed in neural, immune and adipose cells.^48,49^ The retention of an intronic sequence within mRNA can lead to multiple fates for the transcript (Fig. 1B–G), which is to some degree determined by its localization within the cell. IRTs are often removed or degraded to prevent their translation,^50,51^ making them largely undetectable by traditional experimental approaches. However, those that persist can localize in the nucleus, referred to as nuclear intron-retaining transcripts (nIRTs), or be stably expressed in the cytoplasm, termed cytoplasmic intron-retaining transcripts (cIRTs), each exhibiting distinct functions and characteristics.

Fate mapping experiments have revealed a substantial range in the stability of IRTs, with some taking days to reach their fate.^52^ Furthermore, transcripts with retained introns are reportedly less stable, suggesting a coupling between IR and degradation in regulating gene expression.^45^ Notably, there are studies that suggest a more direct link between IRTs and their involvement in transcriptional regulation^53,54^ and cotranscriptional splicing regulation^55^ (Fig. 1D).

### Nuclear functions of IRTs

IRTs have been observed to function as nuclearly detained ‘poised’ transcripts that are then post-transcriptionally spliced in response to a specific stimulus (Fig. 1B).^23^ Transcripts with a high abundance of retained introns seem to be predominantly localized in the nucleus, suggesting that nuclear detention of incompletely spliced transcripts serves as a post-transcriptional mechanism for limiting the expression of mRNAs that would otherwise possess translation potential in the cytoplasm.^56^ One such example is the nuclearly pooled Cdc-like Kinases 1 and 4 (Clk1/4) IRT, which undergoes stress-specific splicing, enabling its export and translation, which in turn permits phosphorylation of the Serine/Arginine-rich (SR) family of splicing factors.^57^ Phosphorylated SR factors can then undergo nuclear import and contribute to spliceosome assembly.^58^ However, such a ‘reservoir’ model, where a precursor pool of IRTs undergoes post-transcriptional splicing, does not apply in all cases and is transcript/context dependent.^59^ Indeed, IRTs can also serve to simply downregulate expression of the cognate canonically spliced transcript, as alluded to above. The degradation of IRTs in the nucleus might occur through the exosome machinery^60^ (Fig. 1C). A notable example is the RBP fused in sarcoma (FUS), which forms an autoregulatory loop through intron retention, with transcripts detained in the nucleus to reduce the amount of cytoplasmic mRNA available for translation.^61^ A recent study considerably extends these findings and demonstrates that FUS IR also fulfils the reservoir model introduced above.^62^ However, it is noteworthy that this class of IRTs that conform to the ‘reservoir’ model represent distinct, transcript- and context-specific outcomes, and do not represent a universal ′logic' for all IRTs.

### Cytoplasmic functions of IRTs

RNA degradation in the cytoplasm occurs through various decay pathways, including a process termed nonsense-mediated mRNA decay (NMD), which plays a crucial role in maintaining cellular homeostasis and clearing defective RNA species^60^ (Fig. 1E). This NMD-mediated degradation process is initiated by the presence of a premature termination codon (PTC) in the transcript, often located within the sequence of the retained intron(s). In this way, the presence of IRTs in the cytoplasm can initiate the NMD cascade, ultimately leading to degradation of those IRTs, and further regulation of gene expression.^63^ AS-NMD coupling represents a conserved regulatory mechanism in physiology. For example, it is paramount in the normal generation of certain haematological constituents including neutrophils, eosinophils and basophils.^44^ Post-transcriptional splicing and AS-NMD coupling of IRTs are comprehensively reviewed elsewhere.^18,20^ Although most cIRTs are rapidly degraded, and therefore should not be detected by RNA sequencing, the stable cytoplasmic localization of some intronic sequences in neurons has been reported since 2013.^64^ Furthermore, we detected that cIRTs undergo tight temporal regulation during human motor neurogenesis.^49^ In this context, cIRTs can coordinate the regulation of gene expression programmes in a spatiotemporally regulated manner, as demonstrated for mouse cortical neurons undergoing synaptic stimulation.^65^ Widespread cIRTs may affect or even overwhelm the efficiency of the NMD quality control system, thereby impairing its ability to degrade defective RNA species and leading to accumulation of ′faulty′ transcripts.^66^ cIRTs are able to evade the NMD surveillance pathway, potentially through concealing the retained introns by protein interactions or altered signals that target particular RNAs to NMD.^67^ Alternatively, since NMD is a translation-dependent process and stress can induce translational arrest, IRTs targeted by NMD may temporarily evade degradation under stress or specific stimuli, potentially gaining translational capacity in a context-dependent manner (Fig. 1E and F). For example, an IRT of the Arc gene, which contains two introns in its 3′ UTR, localizes to dendrites upon neuronal activation. Following stimulation, the IRT is translated before undergoing NMD.^68^ Another example of this occurs during axon guidance; specifically, the alternatively spliced isoform of Roundabout Guidance Receptor 3 (Robo3.2) transcript in mice contains a retained intron bearing an in-frame NMD-inducing PTC. However, the transcript can also lead to the production of a ROBO3.2 protein isoform only when crossing ventral midline structures, due to local translation initiated by floor plate signals.^69^ This spatially regulated control of protein expression allows fine control of commissural axonal trajectory. These examples are reviewed in detail elsewhere.^70^

Intron retention has also been shown to contribute to regulation of protein diversity in neurons by influencing exon inclusion. For example, by directing inclusion of the stress axis–regulated exon (STREX) to a subset of calcium-activated big potassium channel (BKCa) mRNAs, cIRTs coupled with proposed cytoplasmic splicing serve to increase diversity of BKCa splice variants in hippocampal neurons.^71^ The controversial possibility of cytoplasmic splicing is addressed in Box 3. Beyond translation or generation of protein diversity, IRTs in the cytoplasm may be stabilized for other potential functions, which are elaborated below (Fig. 1G).

Box 3 The controversy of cytoplasmic splicing

The possibility of cytoplasmic splicing remains a highly controversial issue. The spliceosome is divided into the major and minor complex. The former is accepted to exert its function in the nucleus. There is also evidence that the minor complex functions predominantly in the nucleus,^72,73^ though one study reported a cytoplasmic presence.^74^ Both major and minor introns have been reported to undergo retention in a functionally important manner.^17,75^ Notably, both protein and RNA constituents of the spliceosome have been reported in primary rat neuronal projections, where isolated dendrites (physically separated from the nucleus), were reportedly capable of splicing.^76^ There are examples of cytoplasmic splicing that are well established, including that of IL1b cytoplasmic intron retaining transcripts (cIRTs), which accumulate in pro-platelet projections and persist into maturation, avoiding conventional splicing and NMD. Upon platelet activation, these IL1b cIRTs are spliced in the anuclear platelet and generate their cognate IL1b mRNA for translation.^77^ Furthermore, the example of pre-mRNA of X-box binding protein 1 (XBP-1) in the unfolded protein response (UPR)^78^ is noteworthy here. The accumulation of incorrectly folded proteins triggers the UPR, which signals through endoplasmic reticulum transmembrane receptors and activates transcription factor 6 (ATF6), which in turn triggers the downstream expression of (spliced) XBP-1. Activation of inositol requiring kinase 1 (IRE1) during UPR enzymatically catalyzes the splicing of a short intron from XBP-1 mRNA through the endoribonuclease activity of IRE1 itself. The resulting XBP-1 protein is a transcription factor that orchestrates various chaperones and protein degradation factors.^79^ This mechanism of cytoplasmic splicing was found to be non-canonical (it relies upon tRNA ligase and an endonuclease, and is spliceosome independent)^80^ and may bear relevance for other cIRTs, though more direct evidence of this is required.

## Non-canonical roles of intron retention

### Regulation of nucleocytoplasmic localization of RBPs

The predominantly cytoplasmic endoribonuclease RNase L is activated by the innate immune response to degrade host and viral RNA, a response designed to reduce viral gene expression. This natural human defence mechanism can be harnessed to serve as an invaluable paradigm to understand the role of RNA in regulating the compartmental distribution of bound RBPs. Indeed, a recent study has demonstrated that upon RNase L activation and degradation of cytoplasmic RNAs, RBPs translocate from the cytoplasm to the nucleus, likely driven by the relative abundance of intact nuclear RNA. Noting the temporally coincident RNA processing changes in the nucleus, this redistribution of RBPs is likely to be functionally consequential.^81^ These data suggest that the nucleocytoplasmic localization of RBPs is in part determined by RNA target availability, at least in some circumstances. Therefore, by extension, the processes of transcription, RNA stability, RNA export and compartment-specific RNA decay will each contribute to determining RBP localization.

**Figure 2:**
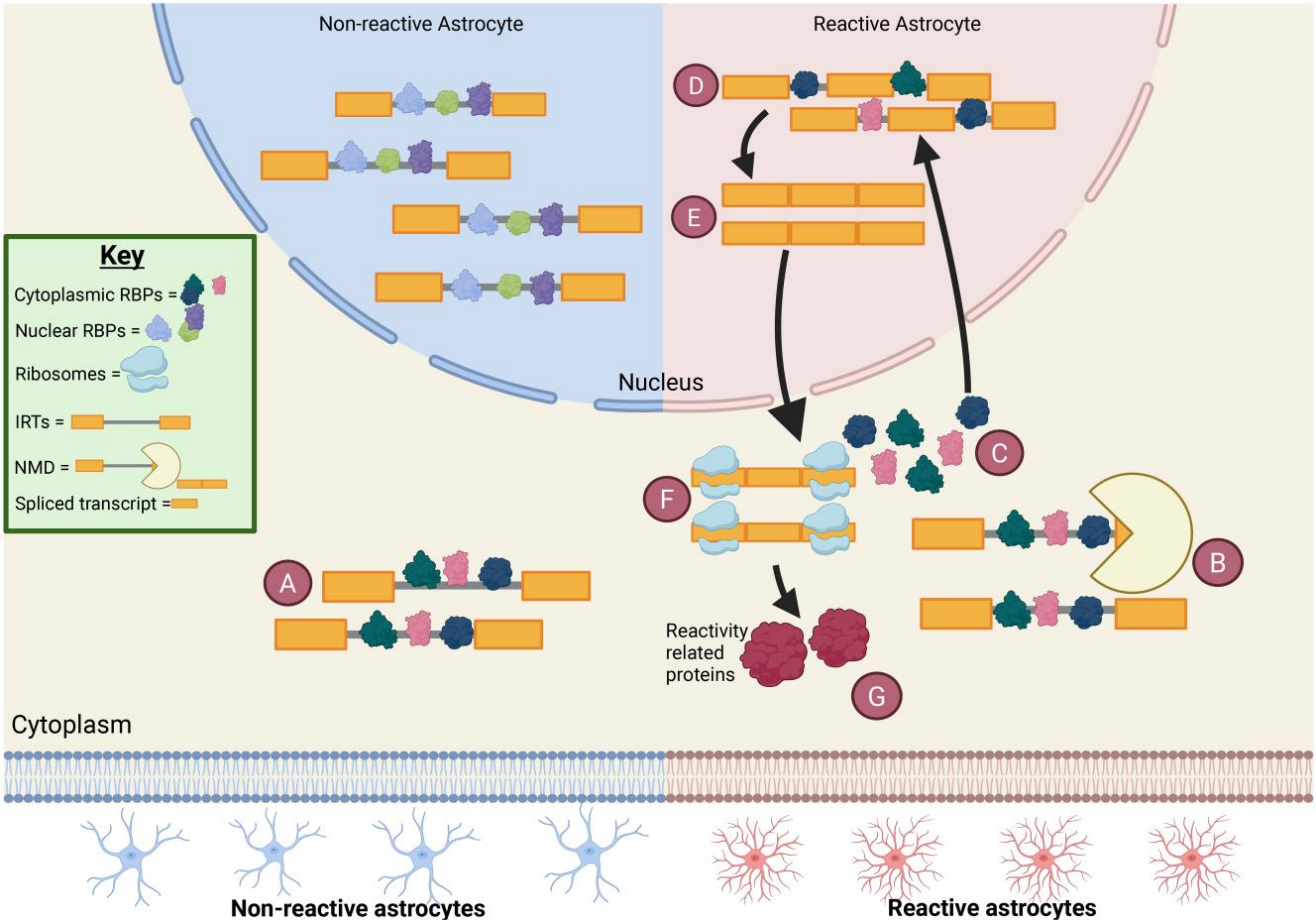
**Intron retaining transcripts may coordinate nucleocytoplasmic localization of RBPs.** Physiological cIRTs exist in the cytoplasm of astrocytes (**A**), which are significantly decreased in ALS astrocytes.^82^ cIRTs may function in physiology to ‘store’ RBPs. Working model: (i) ALS ‘stress’ → (ii) cIRT degradation (**B**) → (iii) liberation of RBPs bound to cIRTs (**C**) → (iv) nuclear import of these RBPs → (v) enhanced splicing of reactivity-related transcripts (**E** and **F**) → (vi) nuclear export and translation into reactivity-related proteins (**F** and **G**) → (vii) reactive transformation of astrocytes. ALS = amyotrophic lateral sclerosis; cIRT = cytoplasmic intron retaining transcript; RBP = RNA-binding protein. Created in BioRender. Wang, C. (2026) https://BioRender.com/r4oepav.

Increasing recognition of cell type-specific RNP expression argues for caution when generalizing from public datasets to more specific experimental contexts. For example, we have previously also reported physiological cIRTs in the cytoplasm of astrocytes (Fig. 2A), which are significantly decreased in ALS astrocytes.^82^ One possible explanation for this finding presupposes that cIRTs may function to sequester RBPs from the nucleus or to serve as a ‘poised’ reservoir of extra-nuclear RBPs in the event they are needed to respond to a particular stress. Together with the decrease in cIRTs, we also noted an increase in NMD activity, which when taken together raise an intriguing hypothesis supporting the following event line: (i) stress → (ii) cIRT degradation (Fig. 2B) → (iii) liberation of RBPs bound to cIRTs (Fig. 2C) → (iv) nuclear import of these RBPs → (v) enhanced splicing of reactivity related transcripts (Fig. 2D and E) → (vi) nuclear export and translation into reactivity-related proteins (Fig. 2F and G) → (vii) reactive transformation of astrocytes. A similar principle may apply to the liberation of RBPs with localized functions following a stress that activates NMD in subcellular compartments of neurons, for example.

### Regulation of miRNAs

Several potential additional roles of IRTs have been speculated.^19^ For example, it has been suggested that IRTs might regulate non-coding RNAs contained within introns. IRTs could interplay with both the canonical and non-canonical pathways for miRNA processing. By the non-canonical pathway, miRNAs can be produced from intronic by-products of splicing which form lariat structures that are subsequently debranched and processed. As such, IRTs that are degraded could reduce the production of these, or if stable, remove a source of miRNA synthesis. Retained introns could also act in competition with miRNA targets to divert miRNA binding and might contain miRNA response elements. Indeed, miRNA putative binding sites are enriched in retained introns compared with non-retained introns.^83^ A reduction in small nucleolar RNAs (snoRNAs) could also represent a possible consequence of intron retention, since these are processed from excised introns.^84^

Although stable cIRTs were reported over a decade ago,^64^ their possible role(s) remain relatively understudied. Data from 2011 showed that intron sequences are retained in a subset of dendritically targeted mRNAs. The retained introns contained ID elements, a class of Short Interspersed Nuclear Element (SINE) retrotransposon, which were shown to be the dendrite-localizing mechanism.^85^ Our group studied time-resolved nucleocytoplasmic dynamics during human motor neurogenesis and demonstrated that IRTs are abundant and spatiotemporally regulated during motor neuron development. We found that cIRTs are detected in up to 13% (> 2000) of the expressed genes. Moreover, we identified a particular class of cIRTs, defined by their spatiotemporal expression profile, that do not correlate with reduced expression of their cognate genes but instead exhibit high capacity for RBP and miRNA occupancy, suggesting regulatory roles beyond controlling gene expression^49,83^ (Supplementary Fig. 1).

### Putative relationship to liquid-liquid phase separation

Noting that not all IRTs are degraded^49,64,86^ raises the question of how they remain stable in the nucleus or cytoplasm. Although stability may be linked to the activity of degradative machinery in each compartment, there may additionally be other mechanisms at play involving liquid-liquid phase separation (LLPS). LLPS refers to the condensation of biomolecules into liquid-like non-membrane delimited organelles. LLPS encompasses protein:protein, protein:RNA and RNA:RNA interactions and is the molecular principle governing transient, non-membrane delimited, functional compartmentalization of cellular contents. Tightly regulated prion-like polymerization occurs driven by weak, transient molecular interactions between low complexity domains (LCDs) of RBPs and other multivalent protein or RNA interaction domains.^87^ This is also highly relevant to the aforementioned RNPs, which can phase-separate as part of their functional repertoire.^88^ Indeed, eukaryotic cells possess a plethora of stress-mitigation strategies, which include large changes in the assembly and/or disassembly of RNPs into ‘granules’ or biocondensates. These processes are likely tightly regulated by prion-like polymerization of RNPs (and/or their constituents).^89^ A plausible model is that IRTs form complexes with such biocondensates thereby rendering them relatively stable in either the nucleus or the cytoplasm. Indeed, a previous report demonstrated that intronic sequences have a great affinity for stress granules,^90^ a form of biocondensate. A recent notable study showed that granules may serve more structural roles as molecular ‘plasters’ following organelle damage.^91^ Given their likely close interaction, one possibility is that IRTs contribute to or further mediate this stress granule phenomenon, though this remains speculative and a hypothesis for future research. We have recently reported that splicing factor proline- and glutamine-rich (SFPQ) and an alternative cytoplasm-predominant isoform with context-specific translation potential both demonstrate markedly increased phase separation when exposed to an intronic sequence derived from an annotated SFPQ intron 9 retention event.^92^

There are also emerging lines of evidence that intronic sequences can drive LLPS in the nucleus. A notable example is the Serine/Arginine-rich Splicing Factor 7 (SRSF7). By binding to its pre-mRNA, SRSF7 protein promotes the inclusion of a poison cassette exon (*PCE*), leading to NMD. Conversely, in the context of elevated SRSF7 levels, NMD is inhibited and the translation of two protein ‘halves’, termed split-open reading frames (ORFs), occurs from the bicistronic *SRSF7-PCE* transcript. One half acts to suppress *PCE* inclusion, but promotes IR in the flanking introns, which are enriched with SRSF7 binding sites. Following SRSF7 binding at these intronic sites, it oligomerizes, leading to large nuclear bodies formed through LLPS. These phase-separated nuclear bodies in turn sequester SRSF7 nascent transcripts, thereby preventing their export and translation. This mechanism serves to regulate SRSF7 protein levels. Such split-ORFs appear to be a design principle for the regulation of a subset of RBPs in a context-dependent manner.^93^ A further recent example relates to FUS retained introns 6 and 7, where one intron was shown to have higher phase separation propensity than the other, at least partially caused by differential secondary structure formation of the intronic sequences.^62^ Modifications to RNA are noteworthy in the context of IRTs. One example is the m6A modification, which can regulate the stability,^94^ splicing^95^ and phase separation potential^96^ of the host transcript. These effects are likely to be influenced by factors such as the site, number and nature of modification, as well as the cellular context.

## The potential role of IRTs in addressing the unique homeostatic challenges of neurons

Neurons have a truly unique and remarkable polarized cytoarchitecture, which correlates with mRNAs exhibiting highly distinctive spatial localization patterns,^97,98^ representing the molecular substrate for localized translation.^99^ In some cases, the localization is rather intuitive, for example mRNAs that encode membrane or secretory proteins predominantly localize to the endoplasmic reticulum (ER) membrane. However, the purpose of partitioning for some RNAs within the cell can be more elusive. It is plausible that cIRTs serve to ‘nucleate’ or seed cytoplasmic translationally repressed phase-separated ‘granules’ containing mRNAs, proteins and potentially other cargo during subcellular localization to the dendrites, axon or synapses. Expanding this concept, cIRTs may also functionally sequester cytoplasmic RBPs at specific subcellular sites, poised for liberation and action (Supplementary Fig. 2), for example following a change in phosphorylation status. Such molecular strategies may enable neurons to robustly respond to stressors at sites remote from the soma.

## Aberrant intron retention and nucleocytoplasmic compartmentalization in ALS

The centrality of disordered splicing to ALS pathology has long been apparent given that the best known hallmark of ALS is TDP-43 nuclear to cytoplasmic mislocalization^3^ and consequent nuclear loss of function, which is associated with usage of non-canonical cryptic splice sites, a disease-related mechanism which might ultimately reduce the abundance of correctly spliced mRNAs for translation.^3,100^ This is illustrated by the aberrant splicing of the stathmin-2 (*STMN2*) and *UNC13A* transcripts triggered by TDP-43 depletion in human neurons.^101,102^ However, the conspicuous absence of TDP-43 pathology in ∼3% of ALS cases, those caused by mutations in *SOD1* or *FUS*, despite such cases being clinically indistinguishable from the majority of ALS cases, led us to reason that there remain further unidentified unifying mechanisms of disease, which present across patients independent of specific mutations. We therefore analysed RNA sequencing data from human induced pluripotent stem cell (iPSC)-derived motor neurons from ALS patients carrying mutations in valosin containing protein (*VCP*; an example of TDP-43 proteinopathy positive ALS), *SOD1* and *FUS* genes, and discovered aberrant IR as a molecular feature across these diverse genetic causes of ALS,^49,86,103,104^ subsequently validated in post-mortem tissue from sporadic ALS.^105^

Given the multifarious functions of IR in human physiology, particularly in the nervous system as alluded to above, it follows that perturbations in this tightly regulated process might modulate or contribute to pathogenesis. At least as early as 1998, IR received attention in the study of ALS pathomechanisms. It was observed in a seminal study that patients with sporadic ALS exhibited loss of the astroglial glutamate transporter excitatory amino acid transporter 2 (EAAT2) in the spinal cord and motor cortex, and that the presence of partial intron 7 retention transcripts was associated with the loss of EAAT2 protein in sporadic ALS patient tissue.^106^ However, momentum to pursue this avenue may have been tempered by a subsequent study which found that the aberrant transcripts were present in controls as well as in ALS patients, and furthermore were present in all CNS regions, without an altered ratio of ‘variant’ to ‘normal’ transcripts in ALS patients compared with controls.^107^ Interestingly, this latter study pointed to the likelihood that an exon-9 skipping EAAT2 transcript variant identified by Lin et al. is physiological, speaking to the relevance of splicing in the nervous system, perhaps particularly in the motor system. This controversy subsequently motivated further study of splicing and intron retention in the motor system.

**Figure 3:**
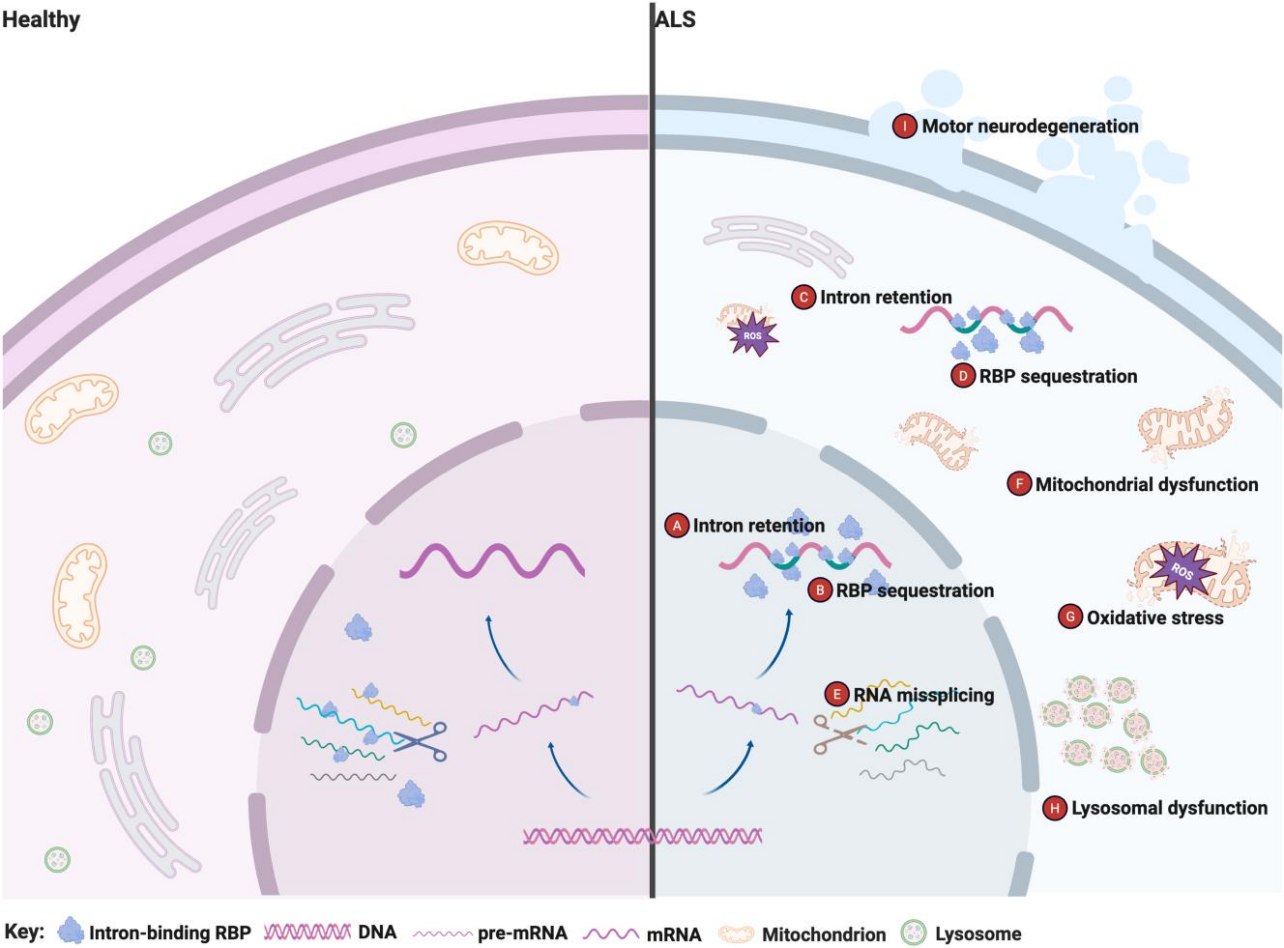
**Working hypothesis of intron-retention related pathogenic cascade in ALS.** In ALS, our working hypothesis is that widespread aberrant intron retention (**A**) may disrupt the function of nuclear RNA binding proteins (RBPs) (**B**). Some intron-retaining transcripts are exported to the cytoplasm (**C**), where they may sequester RBPs (**D**), promoting abnormal liquid-liquid phase separation and aggregation. Disruption of RBP homeostasis may lead to global RNA misprocessing, including dysregulation of splicing in other transcripts (**E**). Together, these molecular disturbances may collectively drive a range of organellar pathophysiology including (but not limited to): mitochondrial impairment (**F**), oxidative stress (**G**), lysosomal dysfunction (**H**) and may ultimately lead to motor neuron degeneration (**I**). ALS = amyotrophic lateral sclerosis. Created in BioRender. Wang, Y. (2026) https://BioRender.com/0353yur.

One plausible explanation for the observed IR in ALS is that it seems to temporally correlate with a global downregulation of splicing components, thereby ‘weakening’ the splicing machinery.^103^ TDP-43 and FUS^108^ as well as SFPQ^109^ are important in the function of the spliceosome, and their mislocalization and malfunction in ALS could lead to IR, initiating a positive feedback cycle. Through subsequent studies, we found evidence to support a model whereby the RBPs SFPQ and FUS bind with high occupancy to retained introns and are mislocalized from the nucleus to the cytoplasm.^103,110^ A putative mechanism underlying this mislocalization of RBPs is that IRTs within the nucleus could sequester and subsequently mislocalize bound RBPs through cytoplasmic export of the complex. However, an alternative scenario is that IRTs and RBPs could move to the cytoplasm independently, subsequently interacting to form an RNP, which may increase the residency time of RBPs in the cytoplasm (Fig. 3).

The aberrant partitioning of RNAs and proteins in ALS may also relate to established perturbations in nucleocytoplasmic transport mechanisms, including the role of nuclear pore components. Indeed, ageing is a major risk factor for ALS and at a cellular level it independently induces changes in nucleocytoplasmic transport. Protein complexes comprising the nuclear pore itself have long half-lives and experience no renewal in postmitotic neurons.^111^ Therefore, an inevitable consequence is that these cells are characterized by increasing mislocalization between the nuclear and cytoplasmic compartments upon ageing.^112^ Nuclear Localization Signal (NLS)-containing proteins employ the importin receptors for transport into the nucleus, machinery that is also impaired upon cellular ageing.^113^ Ageing-related defects in nuclear transport might culminate in the mislocalization and cytoplasmic aggregation of proteins, including RBPs.^114^ It is also established that aged cells accumulate misfolded, aggregated proteins due to compromised protein homeostasis.^115^ It is therefore plausible that cytoplasmic aggregation of RBPs drives a secondary ‘functional’ nuclear transport defect. Indeed, cytoplasmic aggregation of TDP-43 C-terminal fragments can induce mislocalization of certain nuclear pore constituents to the same vicinity.^116^ These pathomechanisms may coexist and occur simultaneously or sequentially. Although this link between ALS and ageing is well recognized (and reviewed elsewhere),^117,118^ the precise mechanistic common denominators remain crucial to resolve in order to potentially inform therapeutic strategy.

## Potential pathogenicity of aberrant intron retention in ALS

We have previously found that hundreds of IRTs exist; >100 IRTs are exported to the cytoplasm^49,86^ in the context of widespread bidirectional mislocalization of both RNAs and proteins between the nucleus and the cytoplasm.^104^ Abnormal cIRTs have been found to be a prominent phenomenon in ALS neurons and are demonstrably increased when compared with healthy controls. Moreover, it has been found that cytoplasmic aberrant IRTs have a strong affinity for RBPs.^86^ These RBPs were reported to form a tightly interconnected network of interacting proteins enriched in mRNA metabolism functions. Significantly, within this network were RBPs known for their involvement in processing capped intron-containing pre-mRNA, implicating disrupted post-transcriptional splicing in ALS pathogenesis. It is noteworthy that this network includes the RBPs SFPQ, TDP-43 and FUS, which are known to exhibit nuclear-to-cytoplasmic mislocalization in ALS.^86^ However, nucleocytoplasmic mislocalization in ALS goes far beyond TDP-43, SFPQ and FUS, encompassing hundreds of mRNAs and proteins.^104^ In ALS iPSC-derived motor neurons carrying mutations in *TARDBP* and *VCP*, hundreds of transcripts and proteins exhibited significant nucleocytoplasmic mislocalization.

In addition, abnormal nIRTs can aggregate into RNA foci that sequester essential RBPs from their physiological functions, further disrupting global RNA metabolism. A salient example is the binding of RBPs to the well-characterized RNA foci observed in Chromosome 9 Open Reading Frame 72 (*C9orf72*) mutant ALS, the most common genetic cause for ALS and FTD. Here, the GGGGCC repeat expansion-containing intron 1-retaining RNA species form nuclear aggregates,^119^ which bind and sequester numerous RBPs including Serine/Arginine-Rich Splicing Factor 2 (SRSF2), Heterogeneous nuclear ribonucleoprotein (hnRNP)A1, hnRNPH/F, Aly/REF export factor (ALYREF),^120^ Adenosine Deaminase RNA Specific B2 (ADARB2)^121^ and HnRNPH.^122^ Interestingly, GGGGCC RNA foci also sequester/co-localize with SFPQ and FUS in cellular models as well as *C9orf72* mutation patient brain tissue.^123^ This is likely to disrupt RNA processing and lead to widespread dysregulation in transcription, splicing and RNA transport. Similar mechanisms have been observed in myotonic dystrophy type 2, where CCUG repeats in the Zinc Finger Protein 9 (*ZNF9*) intron 1 sequester Muscleblind-like (MBNL) RBPs in the muscle cell, primarily within nuclear foci, affecting target gene expression and muscle pathology.^124-126^ Indeed, analogous mechanisms may operate across other repeat expansion disorders, although this has not yet been systematically established.

There are also reports of aberrant intron retention in functionally diverse genes, converging on DNA damage in the context of spinal muscular atrophy (SMA), a type of motor neuron disorder, through R-loop formation.^127^ R-loops are transient RNA-DNA hybrids that form cotranscriptionally, displacing the non-template DNA strand and generating susceptibility to DNA damage, especially if stabilized. The presence of G-rich non-template strands can stabilize R-loops by folding into G-quadruplexes and exacerbating double-strand break formation.^128^ Indeed, DNA damage has been reported as a hallmark feature of ALS and correlates with TDP-43 pathology.^129^

A further mechanism whereby intron retention may contribute to ALS pathology is as a result of translation across retained introns. Retention of introns 3 and 4 in transcripts of *peripherin* leads to the production of a novel, truncated protein isoform of peripherin, a neuronal intermediate filament protein.^130^ Peripherin has previously been implicated in ALS, given its presence in ubiquitinated inclusions, and a recent study suggests peripherin as a novel early diagnostic and prognostic biomarker in ALS.^131^ This truncated isoform is upregulated in ALS patients compared with controls and associates with disease-related inclusions.^130^ In summary, IR represents one among several convergent axes, alongside cryptic splicing, RBP mislocalization, RBP aggregation, nucleocytoplasmic transport defects, mitochondrial dysfunction and axonal transport deficits, and the relative contributions of these mechanisms likely vary by genotype and disease stage.

## Aberrant intron retention and perturbed compartmentalization as therapeutic targets

Broadly, IRTs may lead to a diverse range of functional outcomes in ALS, including: (i) suppression of cellular homeostatic mechanisms; and (ii) expression of factors that increase cellular vulnerability. The crucial next step for clarity in this context is to follow a candidate-based approach and examine the necessity and sufficiency of individual IRTs in ALS pathogenesis. Once an aberrant intron retention event is found to be pathogenic, one potential approach to selectively target these transcripts is the use of antisense oligonucleotides (ASOs), which are short stretches of synthetic DNA that hybridize with complementary RNA through Watson–Crick base pairing. There exist an ever-increasing number of potential chemical modifications to the oligonucleotide exist that allow for efficient target binding with predictable and potent functional outcomes including influencing splicing decisions through blocking RBP binding by steric hindrance on otherwise intact mRNAs. These fully modified ASOs, such as ‘MIXmer’ ASOs, are different from their ‘GAPmer’ ASO counterparts in that they do not activate endogenous RNase H and therefore do not indiscriminately lead to transcript degradation, but can exert a more nuanced intervention, for example influencing a specific splicing decision.

The ability to predictably manipulate the life cycle of targeted RNAs in this manner has already proven transformational in clinical trials for a US Food and Drug Administration (FDA)-approved ASO, nusinersen, in patients with SMA,^132^ which has now entered best practice for the management of SMA. Extrapolating these insights to ALS, where disease-modifying therapeutic options remain extremely limited, could have similar ground-breaking clinical impact. Indeed, a GAPmer approach for targeting SOD1 ALS has already yielded encouraging results in clinical trials in the open label extension phase.^133^ This therapy is FDA and MHRA (Medicines and Healthcare products Regulatory Agency) approved and argues for cautious optimism in adopting antisense approaches for the remaining majority of non-SOD1 ALS patients. It must be noted, however, that while SOD1 and FUS mutations have clear evidence for gain of toxic function amenable to GAPmer approaches, the precise underlying mechanisms are less clear in the remaining 97% of ALS cases, further reinforcing the need for more fundamental discovery. Preventing intron retention using splice-switching ASOs may offer a tractable therapeutic approach as it may then prevent sequestration of their bound RBPs. Translational strategy considerations when targeting ALS-related IRTs include triaging therapeutically tractable events by their significance, abundance, intron length and affinity for binding relevant RBPs. Selectively targeting the IRT isoform should leave the correctly spliced transcripts intact and thereby preserve canonical function. Depending on the approach adopted, some consideration of polytherapy is merited. Perhaps targeting several aberrant transcripts simultaneously may be required to achieve the threshold for cellular rescue. However, recent setbacks in the field underscore the need for cautious framing in this context. For example, the disappointing clinical outcomes of *C9orf72* ASOs (NCT03626012, NCT04931862), which primarily targeted sense repeat transcripts, highlight the complexity of RNA-based pathogenesis in ALS and raise the possibility that effective intervention may require simultaneous targeting of multiple RNA species, including both sense and antisense transcripts, or multiple downstream consequences of RNA dysregulation. Compared with TDP-43 loss-focused strategies targeting single cryptic exon events, such as those in *STMN2* or *UNC13A*, IR-targeting ASOs may present an underexplored opportunity across disease contexts both with and without TDP-43 proteinopathy.

The fact that aberrant IR occurs in ALS prompts consideration of the downstream processing of intronic sequences. Inhibiting the debranching enzyme Dbr, which usually debranches intronic lariats produced during splicing, relieves toxicity secondary to cytoplasmic TDP-43 (or FUS) accumulation. It is likely that the mechanism behind this is sequestration of TDP-43 by the intronic lariats, preventing deleterious interactions with important RNAs and RBPs.^134^ However, the impact of lariats (protective or deleterious) is likely to be context dependent with determinants including cell type, cell substate and disease stage. It is possible that a fine balance exists between detrimental ‘trapping’ of RBPs on retained intronic sequences and a compensatory removal of RBPs that are mislocalized and present in excess. This balance could be deregulated in disease leading to a loss of function of the RBPs bound to the retained intronic sequences. It is possible that the compensatory effects in the acute phase evolve into more maladaptive mechanisms if these are sustained over time and/or occur in the disease context.

Perturbed cellular compartmentalization of RNA and proteins, as reported in ALS, raises the important issue of enhancing nucleocytoplasmic transport integrity as a therapeutic strategy. Indeed, there are a number of studies overexpressing nuclear importins, exportins or transport partners and demonstrating at least partial aggregation reversal of FUS through interaction with their NLSs.^135-137^ It is noteworthy that other studies demonstrate that importins such as Kapβ2/Transportin-1 and Importin-α/β1 can suppress or reverse aberrant phase transitions of additional ALS-linked RBPs, including TATA-Box Binding Protein Associated Factor 15 (TAF15), Ewing Sarcoma breakpoint region 1 (EWSR1), hnRNPA1, hnRNPA2 and TDP-43.^138,139^ Inhibitors of the nuclear export receptor CRM1 (SINE compounds) are also evidently effective in rescuing TDP43-mediated motor phenotypes in different models.^140,141^ Furthermore, overexpression of nucleoporin POM121 was sufficient to rescue dysfunction of TDP-43 targets.^142^ Relocalizing RBPs to the nucleus as a therapeutic strategy might also be realized through small molecular pharmacology. For example, arginine methyltransferase inhibitors in the context of *FUS* mutations mitigate the mislocalization and aggregation of FUS.^143^ Finally, we have reported that treating human ALS motor neurons with VCP D2 ATPase inhibitors largely corrects the observed RNA and protein mislocalization and additionally rescues cellular phenotypes. Importantly, this relocalization of RNAs and proteins also rescues splicing, including partially reversing aberrant intron retention.^104,144^ It is noteworthy that existing referenced data concerning VCP D2 ATPase inhibition derive from iPSC-derived motor neurons and short-term *in vivo* models, and that broader translational generalizability has not yet been established. It is important to recognize that these aforementioned approaches remain at preclinical or at early proof-of-concept stages, with most evidence derived from cellular models and animal systems. Long-term safety, specificity, and efficacy in humans remain unknown and require rigorous evaluation.

## Enhancing discovery of non-canonical RNA functions using artificial intelligence

In recent years, biological research has experienced a paradigm shift, evolving from a traditional hypothesis-driven approach to a sophisticated data-driven methodology. The large volume of sequencing data generated in the last 20 years has motivated the development of a variety of computational techniques to study and characterize the functions and regulations of these RNA sequences.^145-148^ Initially, the data-driven strategy was heavily dependent on manual feature engineering, relying on established knowledge such as gene models or predefined features for tasks like motif enrichment. However, such methods struggle to capture complex, non-linear patterns, emphasize local over global sequence-context relationships and are constrained by prior knowledge, limiting their adaptability to novel or underrepresented biological contexts, such as aberrant intron retention. The emergence of deep learning has marked a significant leap forward, offering a powerful tool for deciphering signal from otherwise complex and noisy biological datasets, and facilitating the unbiased, automated extraction of deep features such as deep regulatory sequences (intronic splicing silencers or intronic splicing enhancers) within introns. Deep learning algorithms have demonstrated the ability to reduce the dimensionality of RNA features and extract more discriminative patterns for predicting RNA-associated interactions,^149-152^ uncovering patterns, relationships and the sophisticated statistical properties of RNA molecules. RNA Large Language Models (LLMs) excel at capturing long-range dependencies and achieving high-level abstraction, making them invaluable for studying RNA function. They have been shown to effectively identify RNA-encoded features and improve predictions of RNA interactions.^149-152^

A key challenge in studying the regulation of non-coding mRNA regions such as retained introns lies in identifying the full repertoire of interacting RBPs, miRNAs and their combined effects on cellular processes in a context-specific manner. In fact, given the context-specific roles of these molecules, it can be posited that the potential functions of RNA molecules are virtually limitless. However, modelling RNA interactions across diverse biological contexts remains difficult for existing algorithms.^153^ Future computational models should generate context-aware RNA representations, allowing for more precise functional predictions. A recent study exemplified this approach by generating contextualized protein interaction networks, producing 394 760 protein representations across 156 cell types from 24 tissues.^153^ Similar advances in RNA modelling could greatly enhance our ability to decipher the regulatory landscape of IRTs (and beyond). Thus, deep learning models applied to RNA biology are poised to revolutionize research, opening a new era of discovery in the field. However, additional work is needed to fully explore the potential of these models in capturing the intricacies of the ‘RNA code’.^154^ Also noteworthy in this context are the limitations of current predictive models, which are typically constrained by training data derived largely from bulk RNA-seq (especially those generated through rRNA-depletion protocols that limit precise identification of intronic reads between mature mRNA and nascent pre-mRNA), a limited array of cell types and incomplete compartment-specific resolution. AI-based predictions of IRTs or regulatory interactions should consequently be interpreted as hypothesis generating until experimentally validated.

## Concluding remarks and future outlook

Intron retention is becoming increasingly recognized as a mechanism to regulate gene expression, and further investigation will be crucial in revealing the extent of the importance of IR in determining RBP function and RBP localization. With interest in studying this form of AS growing, it is important to identify best practice for future studies. Some considerations for RNA sequencing studies such as read depth, library preparation, strandedness, analytical tools and approaches are further considered in Box 4. Beyond such technical considerations to safeguard accuracy of detection, there are further conceptual challenges to overcome. For example, we need to more comprehensively identify the regulators of intron retention beyond cis-features and RBPs to include the influence of epigenetics, epitranscriptomics and transcriptional rate. In order to study the full diversity of IRTs, we need effective tools to block their degradation. Cytoplasmic RNA degradation mechanisms such as NMD can be suppressed genetically [e.g. Up-frameshift 1 (UPF1) knock down] or using small molecules [e.g. SMG1 nonsense-mediated mRNA decay associated PI3K related kinase (SMG1) inhibitors]. The nuclear RNA degradation machinery is less well characterized and requires further study in different cellular and environmental contexts, particularly those of acute versus chronic stress.

Box 4 Technical considerations when studying intron retention

The following points are recommendations to consider when studying intron retention using RNA sequencing. (i) Deep sequencing (ideally 100 M reads; at least 50 M reads). A lower sequencing depth will underestimate intron retention.^155^ (ii) PolyA selection to avoid pre-mRNA contamination. (iii) Strand-specific sequencing to preserve information regarding transcript direction and therefore understanding of overlapping genes and antisense transcripts. (iv) Customized mapping tools: multi-mapping reads and the repetitive sequences within introns would normally lead to these reads being discarded. (v) Ensuring the detection of reads spanning intron-exon junctions to mitigate the risk of erroneously identifying intron-derived non-coding RNA as intron-retaining transcripts (IRTs). (vi) In cases where multiple events within a single transcript are being studied, long read sequencing is advised,^156^ noting the limitations (relatively low depth, high error rates and prone to detecting shorter transcripts). (vii) Extracting IRTs is possible, particularly if they have high phase separation propensity. In this context, heating/shearing steps are necessary for their efficient isolation.^62,157^

Future studies will leverage findings from other fields. For example, studies of intron retention in oncology have found that IRTs exhibit diverse effects, encompassing inhibition of tumour suppressor genes, oncoprotein expression and neoantigen formation. IRTs may also have potential biomarker value and roles in cancer therapy effectiveness and stratification.^158^ Importantly, targeting subsets of functional coherent IRTs seems a tractable therapeutic option across a range of diseases.^158,159^ Many open questions remain regarding the link between intron retention and disease pathogenesis and are outlined in Box 5. In summary, the roles of intron retention in physiology and disease are coming into focus. The confluence of this burgeoning area of molecular biology with an increasing focus on RNA therapeutics, integrated with artificial intelligence approaches, represents an unprecedented opportunity to finally understand and tackle some of the most complex and devastating neurodegenerative disorders such as ALS.

Box 5 Major open questions in intron retention biology

- What are the major roles of stable cytoplasmic intron retaining transcripts? Might these function as an extra-nuclear reservoir of RNA binding proteins?
- How might we identify and understand deep regulatory sequences within retained introns? Could artificial intelligence help to predict these sequences?
- What is the extent of context-specific translation of stable intronic sequences?
- What is the contribution of stable intronic sequences in liquid-liquid phase separation (LLPS) dynamics and vice versa?
- Is aberrant intron retention in disease harmful, compensatory or an interplay of both? Might this vary by disease stage? If aberrant, is targeting one or a small subset of events sufficient to ameliorate disease?
- Might aberrant intron retaining transcripts in disease serve as diagnostic or prognostic biomarkers?

## Supplementary Material

awag142_Supplementary_Data

## References

[1] Taylor JP, Brown RH Jr, Cleveland DW. Decoding ALS: From genes to mechanism. Nature. 2016;539:197–206. 27830784 10.1038/nature20413 PMC5585017

[2] Ripin N, Parker R. Formation, function, and pathology of RNP granules. Cell. 2023;186:4737–4756. 37890457 10.1016/j.cell.2023.09.006 PMC10617657

[3] Neumann M, Sampathu DM, Kwong LK, et al Ubiquitinated TDP-43 in frontotemporal lobar degeneration and amyotrophic lateral sclerosis. Science. 2006;314:130–133. 17023659 10.1126/science.1134108

[4] Ratti A, Buratti E. Physiological functions and pathobiology of TDP-43 and FUS/TLS proteins. J Neurochem. 2016;138(Suppl 1):95–111. 27015757 10.1111/jnc.13625

[5] Balendra R, Sreedharan J, Hallegger M, et al Amyotrophic lateral sclerosis caused by TARDBP mutations: From genetics to TDP-43 proteinopathy. Lancet Neurol. 2025;24:456–470. 40252666 10.1016/S1474-4422(25)00109-7 PMC7617675

[6] Gebauer F, Schwarzl T, Valcárcel J, Hentze MW. RNA-binding proteins in human genetic disease. Nat Rev Genet. 2021;22:185–198. 33235359 10.1038/s41576-020-00302-y

[7] Nabeel-Shah S, Pu S, Burns JD, et al C2H2-zinc-finger transcription factors bind RNA and function in diverse post-transcriptional regulatory processes. Mol Cell. 2024;84:3810–3825.e10. 39303720 10.1016/j.molcel.2024.08.037

[8] Mittal N, Roy N, Babu MM, Janga SC. Dissecting the expression dynamics of RNA-binding proteins in posttranscriptional regulatory networks. Proc Natl Acad Sci USA. 2009;106:20300–20305. 19918083 10.1073/pnas.0906940106 PMC2777960

[9] Saini HK, Griffiths-Jones S, Enright AJ. Genomic analysis of human microRNA transcripts. Proc Natl Acad Sci USA. 2007;104:17719–17724. 17965236 10.1073/pnas.0703890104 PMC2077053

[10] Gao F, Wang F, Chen Y, et al The human genome encodes a multitude of novel miRNAs. Nucleic Acids Res. 2025;53:. 10.1093/nar/gkaf070 PMC11833695 39964476

[11] Brugiolo M, Herzel L, Neugebauer KM. Counting on co-transcriptional splicing. F1000Prime Rep. 2013;5:9. 23638305 10.12703/P5-9 PMC3619158

[12] Carrocci TJ, Neugebauer KM. Emerging and re-emerging themes in co-transcriptional pre-mRNA splicing. Mol Cell. 2024;84:3656–3666. 39366353 10.1016/j.molcel.2024.08.036 PMC11463726

[13] Pandya-Jones A, Bhatt DM, Lin CH, Tong AJ, Smale ST, Black DL. Splicing kinetics and transcript release from the chromatin compartment limit the rate of lipid A-induced gene expression. RNA. 2013;19:811–827. 23616639 10.1261/rna.039081.113 PMC3683915

[14] Khodor YL, Menet JS, Tolan M, Rosbash M. Cotranscriptional splicing efficiency differs dramatically between *Drosophila* and mouse. RNA. 2012;18:2174–2186. 23097425 10.1261/rna.034090.112 PMC3504670

[15] Wang ET, Sandberg R, Luo S, et al Alternative isoform regulation in human tissue transcriptomes. Nature. 2008;456:470–476. 18978772 10.1038/nature07509 PMC2593745

[16] Pan Q, Shai O, Lee LJ, Frey BJ, Blencowe BJ. Deep surveying of alternative splicing complexity in the human transcriptome by high-throughput sequencing. Nat Genet. 2008;40:1413–1415. 18978789 10.1038/ng.259

[17] Braunschweig U, Barbosa-Morais NL, Pan Q, et al Widespread intron retention in mammals functionally tunes transcriptomes. Genome Res. 2014;24:1774–1786. 25258385 10.1101/gr.177790.114 PMC4216919

[18] Monteuuis G, Wong JJL, Bailey CG, Schmitz U, Rasko JEJ. The changing paradigm of intron retention: Regulation, ramifications and recipes. Nucleic Acids Res. 2019;47:11497–11513. 31724706 10.1093/nar/gkz1068 PMC7145568

[19] Wong JJL, Au AYM, Ritchie W, Rasko JEJ. Intron retention in mRNA: No longer nonsense: Known and putative roles of intron retention in normal and disease biology. Bioessays. 2016;38:41–49. 26612485 10.1002/bies.201500117

[20] Jacob AG, Smith CWJ. Intron retention as a component of regulated gene expression programs. Hum Genet. 2017;136:1043–1057. 28391524 10.1007/s00439-017-1791-x PMC5602073

[21] Mattick JS . Introns: Evolution and function. Curr Opin Genet Dev. 1994;4:823–831. 7888751 10.1016/0959-437x(94)90066-3

[22] Amit M, Donyo M, Hollander D, et al Differential GC content between exons and introns establishes distinct strategies of splice-site recognition. Cell Rep. 2012;1:543–556. 22832277 10.1016/j.celrep.2012.03.013

[23] Boutz PL, Bhutkar A, Sharp PA. Detained introns are a novel, widespread class of post-transcriptionally spliced introns. Genes Dev. 2015;29:63–80. 25561496 10.1101/gad.247361.114 PMC4281565

[24] Crerar H, Scott-Solomon E, Bodkin-Clarke C, et al Regulation of NGF signaling by an axonal untranslated mRNA. Neuron. 2019;102:553–563.e8. 30853298 10.1016/j.neuron.2019.02.011 PMC6509357

[25] Sudmant PH, Lee H, Dominguez D, Heiman M, Burge CB. Widespread accumulation of ribosome-associated isolated 3′ UTRs in neuronal cell populations of the aging brain. Cell Rep. 2018;25:2447–2456.e4. 30485811 10.1016/j.celrep.2018.10.094 PMC6354779

[26] Andreassi C, Luisier R, Crerar H, et al Cytoplasmic cleavage of IMPA1 3’ UTR is necessary for maintaining axon integrity. Cell Rep. 2021;34:108778. 33626357 10.1016/j.celrep.2021.108778 PMC7918530

[27] Luisier R, Andreassi C, Fournier L, Riccio A. The predicted RNA-binding protein regulome of axonal mRNAs. Genome Res. 2023;33:1497–1512. 37582635 10.1101/gr.277804.123 PMC10620043

[28] Ma W, Mayr C. A membraneless organelle associated with the endoplasmic reticulum enables 3’UTR-mediated protein-protein interactions. Cell. 2018;175:1492–1506.e19. 30449617 10.1016/j.cell.2018.10.007 PMC6711188

[29] Horste EL, Fansler MM, Cai T, et al Subcytoplasmic location of translation controls protein output. Mol Cell. 2023;83:4509–4523.e11. 38134885 10.1016/j.molcel.2023.11.025 PMC11146010

[30] Luo Y, Pratihar S, Horste EH, et al mRNA interactions with disordered regions control protein activity. *bioRxiv*. [Preprint] doi:10.1101/2023.02.18.529068

[31] Gardner EJ, Nizami ZF, Talbot CC Jr, Gall JG. Stable intronic sequence RNA (sisRNA), a new class of noncoding RNA from the oocyte nucleus of *Xenopus tropicalis*. Genes Dev. 2012;26:2550–2559. 23154985 10.1101/gad.202184.112 PMC3505824

[32] Osman I, Tay MLI, Pek JW. Stable intronic sequence RNAs (sisRNAs): A new layer of gene regulation. Cell Mol Life Sci. 2016;73:3507–3519. 27147469 10.1007/s00018-016-2256-4 PMC11108444

[33] Rasmussen AM, Okholm TLH, Knudsen M, et al Circular stable intronic RNAs possess distinct biological features and are deregulated in bladder cancer. NAR Cancer. 2023;5:zcad041. 37554968 10.1093/narcan/zcad041 PMC10405568

[34] Chan SN, Pek JW. Stable intronic sequence RNAs (sisRNAs): An expanding universe. Trends Biochem Sci. 2019;44:258–272. 30391089 10.1016/j.tibs.2018.09.016

[35] Talhouarne GJS, Gall JG. Lariat intronic RNAs in the cytoplasm of vertebrate cells. Proc Natl Acad Sci USA. 2018;115:E7970–E7977. 30082412 10.1073/pnas.1808816115 PMC6112733

[36] Nostramo RT, Sinopoli PL, Bao A, Metcalf S, Peltier LM, Hopper AK. Free introns of tRNAs as complementarity-dependent regulators of gene expression. Mol Cell. 2025;85:726–741.e6. 39938518 10.1016/j.molcel.2025.01.019 PMC11845289

[37] Chapman KB, Boeke JD. Isolation and characterization of the gene encoding yeast debranching enzyme. Cell. 1991;65:483–492. 1850323 10.1016/0092-8674(91)90466-c

[38] Nam K, Lee G, Trambley J, Devine SE, Boeke JD. Severe growth defect in a *Schizosaccharomyces pombe* mutant defective in intron lariat degradation. Mol Cell Biol. 1997;17:809–818. 9001235 10.1128/mcb.17.2.809 PMC231807

[39] Wilson TJ, Lilley D. RNA catalysis—Is that it? RNA. 2015;21:534–537. 25780127 10.1261/rna.049874.115 PMC4371269

[40] Cech TR, Steitz JA. The noncoding RNA revolution—Trashing old rules to forge new ones. Cell. 2014;157:77–94. 24679528 10.1016/j.cell.2014.03.008

[41] Han P, Chang CP. Long non-coding RNA and chromatin remodeling. RNA Biol. 2015;12:1094–1098. 26177256 10.1080/15476286.2015.1063770 PMC4829272

[42] Statello L, Guo CJ, Chen LL, Huarte M. Gene regulation by long non-coding RNAs and its biological functions. Nat Rev Mol Cell Biol. 2021;22:96–118. 33353982 10.1038/s41580-020-00315-9 PMC7754182

[43] Dominguez D, Tsai YH, Weatheritt R, Wang Y, Blencowe BJ, Wang Z. An extensive program of periodic alternative splicing linked to cell cycle progression. Elife. 2016;5:e10288. 27015110 10.7554/eLife.10288 PMC4884079

[44] Wong JJ, Ritchie W, Ebner OA, et al Orchestrated intron retention regulates normal granulocyte differentiation. Cell. 2013;154:583–595. 23911323 10.1016/j.cell.2013.06.052

[45] Ni T, Yang W, Han M, et al Global intron retention mediated gene regulation during CD4^+^ T cell activation. Nucleic Acids Res. 2016;44:6817–6829. 27369383 10.1093/nar/gkw591 PMC5001615

[46] Forrest ST, Barringhaus KG, Perlegas D, Hammarskjold ML, McNamara CA. Intron retention generates a novel Id3 isoform that inhibits vascular lesion formation. J Biol Chem. 2004;279:32897–32903. 15159391 10.1074/jbc.M404882200

[47] Hadar S, Meller A, Saida N, Shalgi R. Stress-induced transcriptional readthrough into neighboring genes is linked to intron retention. iScience. 2022;25:105543. 36505935 10.1016/j.isci.2022.105543 PMC9732411

[48] Petrova V, Song R, DEEP Consortium, et al Increased chromatin accessibility facilitates intron retention in specific cell differentiation states. Nucleic Acids Res. 2022;50:11563–11579. 36354002 10.1093/nar/gkac994 PMC9723627

[49] Petrić Howe M, Crerar H, Neeves J, et al Physiological intron retaining transcripts in the cytoplasm abound during human motor neurogenesis. Genome Res. 2022;32:1808–1825. 36180233 10.1101/gr.276898.122 PMC9712626

[50] Jaillon O, Bouhouche K, Gout JF, et al Translational control of intron splicing in eukaryotes. Nature. 2008;451:359–362. 18202663 10.1038/nature06495

[51] Gudipati RK, Xu Z, Lebreton A, et al Extensive degradation of RNA precursors by the exosome in wild-type cells. Mol Cell. 2012;48:409–421. 23000176 10.1016/j.molcel.2012.08.018 PMC3496076

[52] Naro C, Jolly A, Di Persio S, et al An orchestrated intron retention program in meiosis controls timely usage of transcripts during germ cell differentiation. Dev Cell. 2017;41:82–93.e4. 28366282 10.1016/j.devcel.2017.03.003 PMC5392497

[53] Pek JW, Osman I, Tay MLI, Zheng RT. Stable intronic sequence RNAs have possible regulatory roles in *Drosophila melanogaster*. J Cell Biol. 2015;211:243–251. 26504165 10.1083/jcb.201507065 PMC4621838

[54] Osman I, Pek JW. A sisRNA/miRNA axis prevents loss of germline stem cells during starvation in *Drosophila*. Stem Cell Reports. 2018;11:4–12. 30008327 10.1016/j.stemcr.2018.06.002 PMC6067505

[55] Chan SN, Pek JW. Distinct biogenesis pathways may have led to functional divergence of the human and *Drosophila* Arglu1 sisRNA. EMBO Rep. 2023;24:e54350. 36533631 10.15252/embr.202154350 PMC9900350

[56] Pimentel H, Parra M, Gee SL, Mohandas N, Pachter L, Conboy JG. A dynamic intron retention program enriched in RNA processing genes regulates gene expression during terminal erythropoiesis. Nucleic Acids Res. 2016;44:838–851. 26531823 10.1093/nar/gkv1168 PMC4737145

[57] Ninomiya K, Kataoka N, Hagiwara M. Stress-responsive maturation of Clk1/4 pre-mRNAs promotes phosphorylation of SR splicing factor. J Cell Biol. 2011;195:27–40. 21949414 10.1083/jcb.201107093 PMC3187705

[58] Roscigno RF, Garcia-Blanco MA. SR proteins escort the U4/U6.U5 tri-snRNP to the spliceosome. RNA. 1995;1:692–706. 7585254 PMC1369311

[59] Pendleton KE, Park SK, Hunter OV, Bresson SM, Conrad NK. Balance between MAT2A intron detention and splicing is determined cotranscriptionally. RNA. 2018;24:778–786. 29563249 10.1261/rna.064899.117 PMC5959247

[60] Yap K, Lim ZQ, Khandelia P, Friedman B, Makeyev EV. Coordinated regulation of neuronal mRNA steady-state levels through developmentally controlled intron retention. Genes Dev. 2012;26:1209–1223. 22661231 10.1101/gad.188037.112 PMC3371409

[61] Humphrey J, Birsa N, Milioto C, et al FUS ALS-causative mutations impair FUS autoregulation and splicing factor networks through intron retention. Nucleic Acids Res. 2020;48:6889–6905. 32479602 10.1093/nar/gkaa410 PMC7337901

[62] Huang WP, Kumar V, Yap K, et al M6A-dependent RNA condensation underlies FUS autoregulation and can be harnessed for ALS therapy development. Sci Adv. 2025;11:eadx1357. 40700505 10.1126/sciadv.adx1357 PMC12285722

[63] Lejeune F, Maquat LE. Mechanistic links between nonsense-mediated mRNA decay and pre-mRNA splicing in mammalian cells. Curr Opin Cell Biol. 2005;17:309–315. 15901502 10.1016/j.ceb.2005.03.002

[64] Khaladkar M, Buckley PT, Lee MT, et al Subcellular RNA sequencing reveals broad presence of cytoplasmic intron-sequence retaining transcripts in mouse and rat neurons. PLoS One. 2013;8:e76194. 24098440 10.1371/journal.pone.0076194 PMC3789819

[65] Mauger O, Lemoine F, Scheiffele P. Targeted intron retention and excision for rapid gene regulation in response to neuronal activity. Neuron. 2016;92:1266–1278. 28009274 10.1016/j.neuron.2016.11.032

[66] Nguyen LS, Wilkinson MF, Gecz J. Nonsense-mediated mRNA decay: Inter-individual variability and human disease. Neurosci Biobehav Rev. 2014;46(Pt 2):175–186. 24239855 10.1016/j.neubiorev.2013.10.016 PMC4021004

[67] Buckley PT, Khaladkar M, Kim J, Eberwine J. Cytoplasmic intron retention, function, splicing, and the sentinel *RNA* hypothesis. Wiley Interdiscip Rev RNA. 2014;5:223–230. 24190870 10.1002/wrna.1203 PMC4449146

[68] Farris S, Lewandowski G, Cox CD, Steward O. Selective localization of *Arc* mRNA in dendrites involves activity- and translation-dependent mRNA degradation. J Neurosci. 2014;34:4481–4493. 24671994 10.1523/JNEUROSCI.4944-13.2014 PMC3965778

[69] Colak D, Ji SJ, Porse BT, Jaffrey SR. Regulation of axon guidance by compartmentalized nonsense-mediated mRNA decay. Cell. 2013;153:1252–1265. 23746841 10.1016/j.cell.2013.04.056 PMC3685487

[70] Petrić Howe M, Patani R. Nonsense-mediated mRNA decay in neuronal physiology and neurodegeneration. Trends Neurosci. 2023;46:879–892. 37543480 10.1016/j.tins.2023.07.001

[71] Bell TJ, Miyashiro KY, Sul JY, et al Intron retention facilitates splice variant diversity in calcium-activated big potassium channel populations. Proc Natl Acad Sci USA. 2010;107:21152–21157. 21078998 10.1073/pnas.1015264107 PMC3000244

[72] Friend K, Kolev NG, Shu MD, Steitz JA. Minor-class splicing occurs in the nucleus of the *Xenopus* oocyte. RNA. 2008;14:1459–1462. 18567814 10.1261/rna.1119708 PMC2491479

[73] Pessa HKJ, Will CL, Meng X, et al Minor spliceosome components are predominantly localized in the nucleus. Proc Natl Acad Sci USA. 2008;105:8655–8660. 18559850 10.1073/pnas.0803646105 PMC2438382

[74] König H, Matter N, Bader R, Thiele W, Müller F. Splicing segregation: The minor spliceosome acts outside the nucleus and controls cell proliferation. Cell. 2007;131:718–729. 18022366 10.1016/j.cell.2007.09.043

[75] Inoue D, Polaski JT, Taylor J, et al Minor intron retention drives clonal hematopoietic disorders and diverse cancer predisposition. Nat Genet. 2021;53:707–718. 33846634 10.1038/s41588-021-00828-9 PMC8177065

[76] Glanzer J, Miyashiro KY, Sul JY, et al RNA splicing capability of live neuronal dendrites. Proc Natl Acad Sci USA. 2005;102:16859–16864. 16275927 10.1073/pnas.0503783102 PMC1277967

[77] Denis MM, Tolley ND, Bunting M, et al Escaping the nuclear confines: Signal-dependent pre-mRNA splicing in anucleate platelets. Cell. 2005;122:379–391. 16096058 10.1016/j.cell.2005.06.015 PMC4401993

[78] Yoshida H . Unconventional splicing of XBP-1 mRNA in the unfolded protein response. Antioxid Redox Signal. 2007;9:2323–2334. 17979529 10.1089/ars.2007.1800

[79] Hetz C, Zhang K, Kaufman RJ. Mechanisms, regulation and functions of the unfolded protein response. Nat Rev Mol Cell Biol. 2020;21:421–438. 32457508 10.1038/s41580-020-0250-z PMC8867924

[80] Back SH, Lee K, Vink E, Kaufman RJ. Cytoplasmic IRE1alpha-mediated XBP1 mRNA splicing in the absence of nuclear processing and endoplasmic reticulum stress. J Biol Chem. 2006;281:18691–18706. 16644724 10.1074/jbc.M602030200

[81] Burke JM, Ripin N, Ferretti MB, et al RNase L activation in the cytoplasm induces aberrant processing of mRNAs in the nucleus. PLoS Pathog. 2022;18:e1010930. 36318584 10.1371/journal.ppat.1010930 PMC9651596

[82] Ziff OJ, Taha DM, Crerar H, et al Reactive astrocytes in ALS display diminished intron retention. Nucleic Acids Res. 2021;49:3168–3184. 33684213 10.1093/nar/gkab115 PMC8034657

[83] Schmitz U, Pinello N, Jia F, et al Intron retention enhances gene regulatory complexity in vertebrates. Genome Biol. 2017;18:216. 29141666 10.1186/s13059-017-1339-3 PMC5688624

[84] Falaleeva M, Stamm S. Processing of snoRNAs as a new source of regulatory non-coding RNAs: snoRNA fragments form a new class of functional RNAs. Bioessays. 2013;35:46–54. 23180440 10.1002/bies.201200117 PMC3732821

[85] Buckley PT, Lee MT, Sul JY, et al Cytoplasmic intron sequence-retaining transcripts can be dendritically targeted via ID element retrotransposons. Neuron. 2011;69:877–884. 21382548 10.1016/j.neuron.2011.02.028 PMC3065018

[86] Tyzack GE, Neeves J, Crerar H, et al Aberrant cytoplasmic intron retention is a blueprint for RNA binding protein mislocalization in VCP-related amyotrophic lateral sclerosis. Brain. 2021;144:1985–1993. 33693641 10.1093/brain/awab078 PMC8370440

[87] Hyman AA, Weber CA, Jülicher F. Liquid-liquid phase separation in biology. Annu Rev Cell Dev Biol. 2014;30:39–58. 25288112 10.1146/annurev-cellbio-100913-013325

[88] Hallegger M, Chakrabarti AM, Lee FCY, et al TDP-43 condensation properties specify its RNA-binding and regulatory repertoire. Cell. 2021;184:4680–4696.e22. 34380047 10.1016/j.cell.2021.07.018 PMC8445024

[89] Riback JA, Katanski CD, Kear-Scott JL, et al Stress-triggered phase separation is an adaptive, evolutionarily tuned response. Cell. 2017;168:1028–1040.e19. 28283059 10.1016/j.cell.2017.02.027 PMC5401687

[90] Martin S, Bellora N, González-Vallinas J, et al Preferential binding of a stable G3BP ribonucleoprotein complex to intron-retaining transcripts in mouse brain and modulation of their expression in the cerebellum. J Neurochem. 2016;139:349–368. 27513819 10.1111/jnc.13768

[91] Bussi C, Mangiarotti A, Vanhille-Campos C, et al Stress granules plug and stabilize damaged endolysosomal membranes. Nature. 2023;623:1062–1069. 37968398 10.1038/s41586-023-06726-w PMC10686833

[92] Neeves J, Petrić Howe M, Ziff OJ, et al An alternative cytoplasmic SFPQ isoform with reduced phase separation potential is up-regulated in ALS. Sci Adv. 2025;11:eadt4814. 40845103 10.1126/sciadv.adt4814 PMC12372870

[93] Königs V, de Oliveira Freitas Machado C, Arnold B, et al SRSF7 maintains its homeostasis through the expression of split-ORFs and nuclear body assembly. Nat Struct Mol Biol. 2020;27:260–273. 32123389 10.1038/s41594-020-0385-9 PMC7096898

[94] Wang X, Lu Z, Gomez A, et al N6-methyladenosine-dependent regulation of messenger RNA stability. Nature. 2014;505:117–120. 24284625 10.1038/nature12730 PMC3877715

[95] Zhou KI, Shi H, Lyu R, et al Regulation of co-transcriptional pre-mRNA splicing by mA through the low-complexity protein hnRNPG. Mol Cell. 2019;76:70–81.e9. 31445886 10.1016/j.molcel.2019.07.005 PMC6778029

[96] Ries RJ, Zaccara S, Klein P, et al Ma enhances the phase separation potential of mRNA. Nature. 2019;571:424–428. 31292544 10.1038/s41586-019-1374-1 PMC6662915

[97] Hüttelmaier S, Zenklusen D, Lederer M, et al Spatial regulation of beta-actin translation by Src-dependent phosphorylation of ZBP1. Nature. 2005;438:512–515. 16306994 10.1038/nature04115

[98] Lécuyer E, Yoshida H, Parthasarathy N, et al Global analysis of mRNA localization reveals a prominent role in organizing cellular architecture and function. Cell. 2007;131:174–187. 17923096 10.1016/j.cell.2007.08.003

[99] Buxbaum AR, Haimovich G, Singer RH. In the right place at the right time: Visualizing and understanding mRNA localization. Nat Rev Mol Cell Biol. 2015;16:95–109. 25549890 10.1038/nrm3918 PMC4484810

[100] Ling JP, Pletnikova O, Troncoso JC, Wong PC. TDP-43 repression of nonconserved cryptic exons is compromised in ALS-FTD. Science. 2015;349:650–655. 26250685 10.1126/science.aab0983 PMC4825810

[101] Klim JR, Williams LA, Limone F, et al ALS-implicated protein TDP-43 sustains levels of STMN2, a mediator of motor neuron growth and repair. Nat Neurosci. 2019;22:167–179. 30643292 10.1038/s41593-018-0300-4 PMC7153761

[102] Brown AL, Wilkins OG, Keuss MJ, et al TDP-43 loss and ALS-risk SNPs drive mis-splicing and depletion of UNC13A. Nature. 2022;603:131–137. 35197628 10.1038/s41586-022-04436-3 PMC8891020

[103] Luisier R, Tyzack GE, Hall CE, et al Intron retention and nuclear loss of SFPQ are molecular hallmarks of ALS. Nat Commun. 2018;9:2010. 29789581 10.1038/s41467-018-04373-8 PMC5964114

[104] Ziff OJ, Harley J, Wang Y, et al Nucleocytoplasmic mRNA redistribution accompanies RNA binding protein mislocalization in ALS motor neurons and is restored by VCP ATPase inhibition. Neuron. 2023;111:3011–3027.e7. 37480846 10.1016/j.neuron.2023.06.019

[105] Hogan AL, Grima N, Fifita JA, et al Splicing factor proline and glutamine rich intron retention, reduced expression and aggregate formation are pathological features of amyotrophic lateral sclerosis. Neuropathol Appl Neurobiol. 2021;47:990–1003. 34288034 10.1111/nan.12749

[106] Lin CL, Bristol LA, Jin L, et al Aberrant RNA processing in a neurodegenerative disease: The cause for absent EAAT2, a glutamate transporter, in amyotrophic lateral sclerosis. Neuron. 1998;20:589–602. 9539131 10.1016/s0896-6273(00)80997-6

[107] Flowers JM, Powell JF, Leigh PN, Andersen P, Shaw CE. Intron 7 retention and exon 9 skipping EAAT2 mRNA variants are not associated with amyotrophic lateral sclerosis. Ann Neurol. 2001;49:643–649. 11357955

[108] Tsuiji H, Iguchi Y, Furuya A, et al Spliceosome integrity is defective in the motor neuron diseases ALS and SMA. EMBO Mol Med. 2013;5:221–234. 23255347 10.1002/emmm.201202303 PMC3569639

[109] Patton JG, Porro EB, Galceran J, Tempst P, Nadal-Ginard B. Cloning and characterization of PSF, a novel pre-mRNA splicing factor. Genes Dev. 1993;7:393–406. 8449401 10.1101/gad.7.3.393

[110] Tyzack GE, Luisier R, Taha DM, et al Widespread FUS mislocalization is a molecular hallmark of amyotrophic lateral sclerosis. Brain. 2019;142:2572–2580. 31368485 10.1093/brain/awz217 PMC6735815

[111] Savas JN, Toyama BH, Xu T, Yates JR 3rd, Hetzer MW. Extremely long-lived nuclear pore proteins in the rat brain. Science. 2012;335:942. 22300851 10.1126/science.1217421 PMC3296478

[112] D’Angelo MA, Raices M, Panowski SH, Hetzer MW. Age-dependent deterioration of nuclear pore complexes causes a loss of nuclear integrity in postmitotic cells. Cell. 2009;136:284–295. 19167330 10.1016/j.cell.2008.11.037 PMC2805151

[113] Mertens J, Paquola ACM, Ku M, et al Directly reprogrammed human neurons retain aging-associated transcriptomic signatures and reveal age-related nucleocytoplasmic defects. Cell Stem Cell. 2015;17:705–718. 26456686 10.1016/j.stem.2015.09.001 PMC5929130

[114] Kim HJ, Taylor JP. Lost in transportation: Nucleocytoplasmic transport defects in ALS and other neurodegenerative diseases. Neuron. 2017;96:285–297. 29024655 10.1016/j.neuron.2017.07.029 PMC5678982

[115] Taylor RC, Dillin A. Aging as an event of proteostasis collapse. Cold Spring Harb Perspect Biol. 2011;3:a004440. 21441594 10.1101/cshperspect.a004440 PMC3101847

[116] Woerner AC, Frottin F, Hornburg D, et al Cytoplasmic protein aggregates interfere with nucleocytoplasmic transport of protein and RNA. Science. 2016;351:173–176. 26634439 10.1126/science.aad2033

[117] Pandya VA, Patani R. Decoding the relationship between ageing and amyotrophic lateral sclerosis: A cellular perspective. Brain. 2020;143:1057–1072. 31851317 10.1093/brain/awz360 PMC7174045

[118] Pandya VA, Patani R. Region-specific vulnerability in neurodegeneration: Lessons from normal ageing. Ageing Res Rev. 2021;67:101311. 33639280 10.1016/j.arr.2021.101311 PMC8024744

[119] Niblock M, Smith BN, Lee YB, et al Retention of hexanucleotide repeat-containing intron in C9orf72 mRNA: Implications for the pathogenesis of ALS/FTD. Acta Neuropathol Commun. 2016;4:18. 26916632 10.1186/s40478-016-0289-4 PMC4766718

[120] Cooper-Knock J, Higginbottom A, Stopford MJ, et al Antisense RNA foci in the motor neurons of C9ORF72-ALS patients are associated with TDP-43 proteinopathy. Acta Neuropathol. 2015;130:63–75. 25943887 10.1007/s00401-015-1429-9 PMC4468790

[121] Donnelly CJ, Zhang PW, Pham JT, et al RNA toxicity from the ALS/FTD C9ORF72 expansion is mitigated by antisense intervention. Neuron. 2013;80:415–428. 24139042 10.1016/j.neuron.2013.10.015 PMC4098943

[122] Lee YB, Chen HJ, Peres JN, et al Hexanucleotide repeats in ALS/FTD form length-dependent RNA foci, sequester RNA binding proteins, and are neurotoxic. Cell Rep. 2013;5:1178–1186. 24290757 10.1016/j.celrep.2013.10.049 PMC3898469

[123] Bajc Česnik A, Darovic S, Prpar Mihevc S, et al Nuclear RNA foci from *C9ORF72* expansion mutation form paraspeckle-like bodies. J Cell Sci. 2019;132:jcs224303. 30745340 10.1242/jcs.224303

[124] Sznajder ŁJ, Thomas JD, Carrell EM, et al Intron retention induced by microsatellite expansions as a disease biomarker. Proc Natl Acad Sci USA. 2018;115:4234–4239. 29610297 10.1073/pnas.1716617115 PMC5910826

[125] Fardaei M, Rogers MT, Thorpe HM, et al Three proteins, MBNL, MBLL and MBXL, co-localize in vivo with nuclear foci of expanded-repeat transcripts in DM1 and DM2 cells. Hum Mol Genet. 2002;11:805–814. 11929853 10.1093/hmg/11.7.805

[126] Liquori CL, Ricker K, Moseley ML, et al Myotonic dystrophy type 2 caused by a CCTG expansion in intron 1 of *ZNF9*. Science. 2001;293:864–867. 11486088 10.1126/science.1062125

[127] Jangi M, Fleet C, Cullen P, et al SMN deficiency in severe models of spinal muscular atrophy causes widespread intron retention and DNA damage. Proc Natl Acad Sci USA. 2017;114:E2347–E2356. 28270613 10.1073/pnas.1613181114 PMC5373344

[128] García-Muse T, Aguilera A. R loops: From physiological to pathological roles. Cell. 2019;179:604–618. 31607512 10.1016/j.cell.2019.08.055

[129] Ziff OJ, Neeves J, Mitchell J, et al Integrated transcriptome landscape of ALS identifies genome instability linked to TDP-43 pathology. Nat Commun. 2023;14:2176. 37080969 10.1038/s41467-023-37630-6 PMC10119258

[130] Xiao S, Tjostheim S, Sanelli T, et al An aggregate-inducing peripherin isoform generated through intron retention is upregulated in amyotrophic lateral sclerosis and associated with disease pathology. J Neurosci. 2008;28:1833–1840. 18287500 10.1523/JNEUROSCI.3222-07.2008 PMC6671437

[131] Bombaci A, Marco GD, Casale F, et al Peripherin: A novel early diagnostic and prognostic plasmatic biomarker in Amyotrophic Lateral Sclerosis. Eur J Neurol. 2025;32:e70241. 40476320 10.1111/ene.70241 PMC12142269

[132] Finkel RS, Chiriboga CA, Vajsar J, et al Treatment of infantile-onset spinal muscular atrophy with nusinersen: A phase 2, open-label, dose-escalation study. Lancet. 2016;388:3017–3026. 27939059 10.1016/S0140-6736(16)31408-8

[133] Miller TM, Cudkowicz ME, Genge A, et al Trial of antisense oligonucleotide tofersen for *SOD1* ALS. N Engl J Med. 2022;387:1099–1110. 36129998 10.1056/NEJMoa2204705

[134] Armakola M, Higgins MJ, Figley MD, et al Inhibition of RNA lariat debranching enzyme suppresses TDP-43 toxicity in ALS disease models. Nat Genet. 2012;44:1302–1309. 23104007 10.1038/ng.2434 PMC3510335

[135] Yoshizawa T, Ali R, Jiou J, et al Nuclear import receptor inhibits phase separation of FUS through binding to multiple sites. Cell. 2018;173:693–705.e22. 29677513 10.1016/j.cell.2018.03.003 PMC6234985

[136] Hofweber M, Hutten S, Bourgeois B, et al Phase separation of FUS is suppressed by its nuclear import receptor and arginine methylation. Cell. 2018;173:706–719.e13. 29677514 10.1016/j.cell.2018.03.004

[137] Qamar S, Wang G, Randle SJ, et al FUS phase separation is modulated by a molecular chaperone and methylation of arginine cation-π interactions. Cell. 2018;173:720–734.e15. 29677515 10.1016/j.cell.2018.03.056 PMC5927716

[138] Guo L, Kim HJ, Wang H, et al Nuclear-import receptors reverse aberrant phase transitions of RNA-binding proteins with prion-like domains. Cell. 2018;173:677–692.e20. 29677512 10.1016/j.cell.2018.03.002 PMC5911940

[139] Khalil B, Chhangani D, Wren MC, et al Nuclear import receptors are recruited by FG-nucleoporins to rescue hallmarks of TDP-43 proteinopathy. Mol Neurodegener. 2022;17:80. 36482422 10.1186/s13024-022-00585-1 PMC9733332

[140] Chou CC, Zhang Y, Umoh ME, et al TDP-43 pathology disrupts nuclear pore complexes and nucleocytoplasmic transport in ALS/FTD. Nat Neurosci. 2018;21:228–239. 29311743 10.1038/s41593-017-0047-3 PMC5800968

[141] Archbold HC, Jackson KL, Arora A, et al TDP43 nuclear export and neurodegeneration in models of amyotrophic lateral sclerosis and frontotemporal dementia. Sci Rep. 2018;8:4606. 29545601 10.1038/s41598-018-22858-w PMC5854632

[142] Rothstein JD, Keeley O, Warlick C, et al Sporadic ALS induced pluripotent stem cell derived neurons reveal hallmarks of TDP-43 loss of function. Nat Commun. 2025;16:7092. 40753166 10.1038/s41467-025-62482-7 PMC12318032

[143] Fujii S, Takanashi K, Kitajo K, Yamaguchi A. Treatment with a global methyltransferase inhibitor induces the intranuclear aggregation of ALS-linked FUS mutant in vitro. Neurochem Res. 2016;41:826–835. 26603295 10.1007/s11064-015-1758-z

[144] Harley J, Hagemann C, Serio A, Patani R. TDP-43 and FUS mislocalization in VCP mutant motor neurons is reversed by pharmacological inhibition of the VCP D2 ATPase domain. Brain Commun. 2021;3:fcab166. 34396115 10.1093/braincomms/fcab166 PMC8361416

[145] Si J, Cui J, Cheng J, Wu R. Computational prediction of RNA-binding proteins and binding sites. Int J Mol Sci. 2015;16:26303–26317. 26540053 10.3390/ijms161125952 PMC4661811

[146] Puton T, Kozlowski L, Tuszynska I, Rother K, Bujnicki JM. Computational methods for prediction of protein–RNA interactions. J Struct Biol. 2012;179:261–268. 22019768 10.1016/j.jsb.2011.10.001

[147] Agarwal A, Kant S, Bahadur RP. Efficient mapping of RNA-binding residues in RNA-binding proteins using local sequence features of binding site residues in protein-RNA complexes. Proteins: Structure, Function, and Bioinformatics. 2023;91:1361–1379. 10.1002/prot.26528 37254800

[148] Yan Y, Li W, Wang S, Huang T. Seq-RBPPred: Predicting RNA-binding proteins from sequence. ACS Omega. 2024;9:12734–12742. 38524500 10.1021/acsomega.3c08381 PMC10955590

[149] Pan X, Fan YX, Yan J, Shen HB. IPMiner: Hidden ncRNA-protein interaction sequential pattern mining with stacked autoencoder for accurate computational prediction. BMC Genomics. 2016;17:582. 27506469 10.1186/s12864-016-2931-8 PMC4979166

[150] Penić RJ, Vlašić T, Huber RG, Wan Y, Šikić M. RiNALMo: General-purpose RNA language models can generalize well on structure prediction tasks. Nat Commun. 2025;16:5671. 40593636 10.1038/s41467-025-60872-5 PMC12219582

[151] Zhang Y, Lang M, Jiang J, et al Multiple sequence alignment-based RNA language model and its application to structural inference. Nucleic Acids Res. 2024;52:e3. 37941140 10.1093/nar/gkad1031 PMC10783488

[152] Yin W, Zhang Z, He L, et al ERNIE-RNA: An RNA language model with structure-enhanced representations. Nat Commun. 2025;16:10076. 41253752 10.1038/s41467-025-64972-0 PMC12627772

[153] Li MM, Huang Y, Sumathipala M, et al Contextual AI models for single-cell protein biology. Nat Methods. 2024;21:1546–1557. 39039335 10.1038/s41592-024-02341-3 PMC11310085

[154] Jung V, Vincent-Cuaz C, Tumescheit C, et al Decoding the interactions and functions of non-coding RNA with artificial intelligence. Nat Rev Mol Cell Biol. 2025;26:797–818. 40537558 10.1038/s41580-025-00857-w

[155] Dvinge H, Bradley RK. Widespread intron retention diversifies most cancer transcriptomes. Genome Med. 2015;7:45. 26113877 10.1186/s13073-015-0168-9 PMC4480902

[156] Lorenzi C, Barriere S, Arnold K, Luco RF, Oldfield AJ, Ritchie W. IRFinder-S: A comprehensive suite to discover and explore intron retention. Genome Biol. 2021;22:307. 34749764 10.1186/s13059-021-02515-8 PMC8573998

[157] Zeng C, Chujo T, Hirose T, Hamada M. Landscape of semi-extractable RNAs across five human cell lines. Nucleic Acids Res. 2023;51:7820–7831. 37463833 10.1093/nar/gkad567 PMC10450185

[158] Smart AC, Margolis CA, Pimentel H, et al Intron retention is a source of neoepitopes in cancer. Nat Biotechnol. 2018;36:1056–1058. 30114007 10.1038/nbt.4239 PMC6226333

[159] Braun CJ, Stanciu M, Boutz PL, et al Coordinated splicing of regulatory detained introns within oncogenic transcripts creates an exploitable vulnerability in malignant glioma. Cancer Cell. 2017;32:411–426.e11. 28966034 10.1016/j.ccell.2017.08.018 PMC5929990

