# Supplementary material for: Intron retention in health and amyotrophic lateral sclerosis
Source: Brain. 2026 Aug 3;149(8):2604–18. doi: 10.1093/brain/awag142 (PMC13431790; doi:10.1093/brain/awag142)
Supplement: awag142_Supplementary_Data [file awag142_supplementary_data.pdf]

Supplementary Figures

Supplementary Figure 1. Intron retaining transcripts may function to regulate RBP availability and miRNA activity. Intron retaining transcripts (IRTs) may be stabilised and serve to i) sequester RBPs, thereby leading to a loss of RBP function (A). IRTs can also bind and regulate (or indeed be regulated by) miRNAs (B). Created in BioRender. Wang, C. (2026) https://BioRender.com/bgbfy2m.

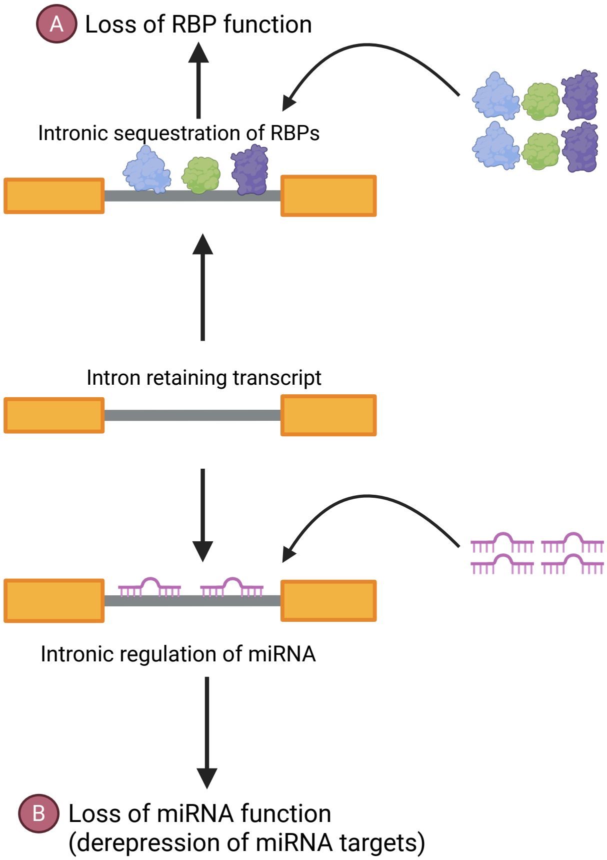
A
Loss of RBP function
Intronic sequestration of RBPs
Intron retaining transcript
Intronic regulation of miRNA
B
Loss of miRNA function
(derepression of miRNA targets)

Supplementary Figure 2. Distributed and translationally repressed ribonucleoproteins help to achieve and maintain homeostasis in highly polarised neurons. Working model: cytoplasmic intron retaining transcripts (cIRTs) serve to ‘nucleate’ or seed cytoplasmic translationally-repressed phase separated ‘granules’ containing mRNAs, proteins and potentially other cargo during subcellular localisation to the dendrites, axon or synapses. Created in BioRender. Wang, C. (2026) https://BioRender.com/k5gr7a5.

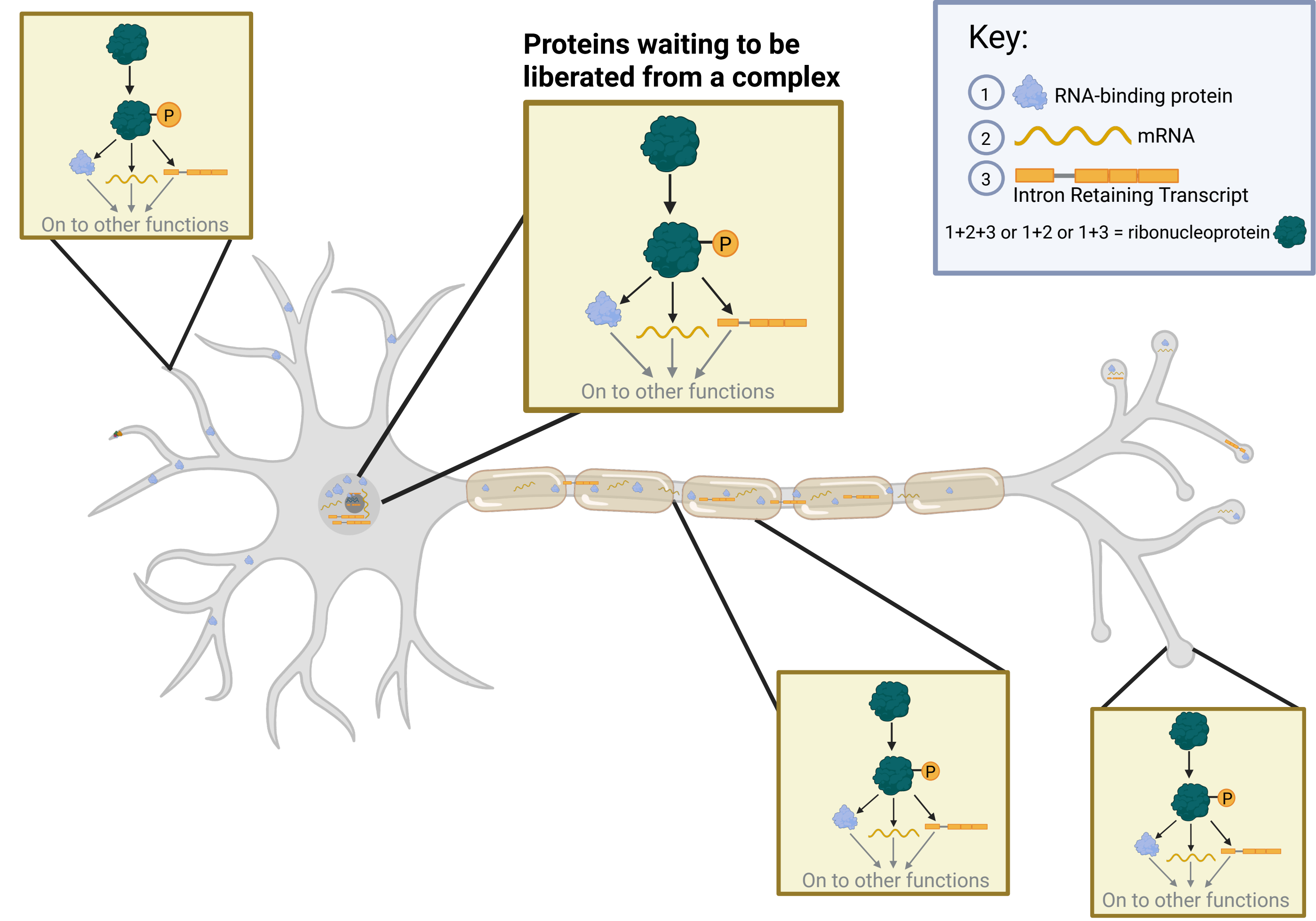

Proteins waiting to be liberated from a complex
Key:
1 RNA-binding protein
2 mRNA
3 Intron Retaining Transcript
1+2+3 or 1+2 or 1+3 = ribonucleoprotein
P
On to other functions
On to other functions
On to other functions
On to other functions
On to other functions
